# Care Continuity, Nephrologists’ Dialysis Facility Preferences, and Outcomes

**DOI:** 10.1001/jamahealthforum.2025.0423

**Published:** 2025-04-11

**Authors:** Eugene Lin, Khristina I. Lung, Derick Rapista, Leane S. Kuo, Darius Lakdawalla, Desi Peneva, Karen Van Nuys

**Affiliations:** 1 Department of Medicine, Division of Nephrology, Keck School of Medicine of the University of Southern California, Los Angeles; 2 Leonard D. Schaeffer Center for Health Policy & Economics, University of Southern California, Los Angeles; 3 Department of Health Policy and Management, Sol Price School of Public Policy, University of Southern California, Los Angeles; 4 Department of Population and Public Health Sciences, Keck School of Medicine of the University of Southern California, Los Angeles; 5 Department of Pharmaceutical and Health Economics, Alfred E. Mann School of Pharmacy and Pharmaceutical Sciences, University of Southern California, Los Angeles

## Abstract

**Importance:**

Patients may initiate dialysis at their predialysis nephrologists’ primary facilities (ie, where the nephrologist saw the most patients) to preserve continuity of care, even if the facilities are of low quality. Patients from minoritized racial and ethnic groups may be the most negatively impacted.

**Objective:**

To examine starts at nephrologists’ primary facilities, downstream outcomes, and racial disparities in dialysis start quality.

**Design, Setting, and Participants:**

This cohort study used Medicare administrative data of patients initiating dialysis at freestanding US dialysis facilities from January 1, 2015, to October 31, 2020, with 1 year of follow-up (ending October 31, 2021). Analyses concluded January 26, 2025. Participants were adults with fee-for-service Medicare initiating dialysis.

**Exposures:**

Quality of nephrologists’ primary facilities (using publicly available 5-star ratings) and primary facilities’ proximity to patients.

**Main Outcomes and Measures:**

The primary outcomes were starting dialysis at the nephrologist’s primary facility (ie, primary facility starts), whether the starting facility was high quality (ie, 4-star or 5-star ratings), mortality and hospitalization rates, and racial and ethnic disparities in high-quality primary facilities and in starting dialysis at high-quality facilities. Analyses used multivariable linear and Poisson regression with hospital service area fixed effects (unique intercepts for each area).

**Results:**

Of 143 776 adults (median [IQR] age, 73 [67-79] years; 64 447 female [45%]; 4989 Asian [3%]; 28 515 Black [20%]; 11 296 Hispanic [8%]; 96 639 non-Hispanic White [67%]), 64 186 (45%) had managing nephrologists with high-quality primary facilities. Primary facility starts were lower as the primary facility’s quality increased (0.5 percentage points [pp] lower for every 1-star increase in rating; 95% CI, 0.1-0.8 pp; *P* = .03). In contrast, primary facility starts were 33.9 pp (95% CI, 33.0-34.9 pp; *P* < .001) more likely when primary facilities were close to patients than when distant. Each additional quality star in nephrologists’ primary facility was associated with more 4-star or 5-star facility starts (7.4 pp; 95% CI, 6.9-7.9 pp) and 4.5 fewer hospitalizations per 100 person-years (95% CI, 2.8-6.1 hospitalizations per 100 person-years). Compared with White patients, Black patients were 2.8 pp (95% CI, 1.7-3.9 pp) less likely to start at 4-star or 5-star facilities and 2.0 pp (95% CI, 1.0-3.0 pp) less likely to be treated by nephrologists with 4-star or 5-star primary facilities.

**Conclusions and Relevance:**

Primary facility starts were common even when they were low quality, and outcomes were worse when nephrologists had low-quality primary facilities. Black patients disproportionately start dialysis at low-quality facilities and are less likely to have nephrologists with high-quality primary facilities. Policies that promote improved access to high-quality dialysis facilities may be necessary to alleviate these disparities.

## Key Points

**Question:**

Is care continuity with the predialysis nephrologist associated with dialysis start quality?

**Findings:**

In this cohort study of 143 776 adults, patients with fee-for-service Medicare often started dialysis at their nephrologists’ primary facilities (ie, where nephrologists spent the most time) even when they were low quality. Patients had significantly higher hospitalization rates when their nephrologists had low-quality primary facilities, and Black patients more often had nephrologists with low-quality primary facilities and had lower quality dialysis starts.

**Meaning:**

These findings suggest that a nephrologist’s primary facility is associated with dialysis start quality and that there are prominent racial disparities in access to nephrologists with high-quality primary facilities; policies that promote improved access to high-quality dialysis facilities may be necessary to alleviate these disparities.

## Teaser

This cohort study analyzes whether care continuity with the predialysis nephrologist is associated with dialysis start quality, downstream outcomes associated with facility quality, and racial disparities in dialysis start quality.

## Introduction

Patients receiving dialysis for end-stage kidney disease (ESKD) have a 20% 1-year mortality rate and are hospitalized 1.5 times per year.^1^ Initiating dialysis is especially rife with poor outcomes.^2^

Unfortunately, research is sparse on how patients choose dialysis facilities and whether patients prioritize care continuity with the nephrologist, travel distance, or facility quality. Although the first 2 are transparent to patients, the third is opaque. To help patients identify high-quality facilities, the Centers for Medicare & Medicaid Services (CMS) publishes 5-star quality ratings through Dialysis Facility Compare (DFC). However, it is unclear whether DFC ratings are effective, and patients with poor health literacy or of low socioeconomic status may not benefit.^3,4^

Instead of picking high-quality facilities, patients might select facilities where their nephrologist sees patients. In non-ESKD settings, care continuity results in lower mortality^5,6,7^ and improved care efficiency.^8^ Care continuity in dialysis, however, is not well studied,^9,10^ especially during the transition to dialysis. Patients may be negatively impacted by care continuity if their nephrologists primarily manage patients at low-quality facilities.

Poor care continuity likely contributes to racial and ethnic disparities in non-ESKD settings.^11,12,13^ It is unclear whether patients from minoritized racial and ethnic groups initiating dialysis are also less likely to retain their nephrologist. Even if they do, care continuity could exacerbate disparities in dialysis quality if such patients are more likely treated by nephrologists at low-quality facilities. A previous study^14^ suggested that Black patients initiate dialysis at lower-quality facilities even when higher-quality ones are closer but did not examine the patient-nephrologist relationship or downstream outcomes.

In this study, we examined whether fee-for-service (FFS) Medicare patients were more likely to start dialysis at facilities where their predialysis nephrologist primarily managed dialysis (primary facilities), at high-quality facilities, or at nearby facilities. We further examined outcomes associated with the quality of nephrologists’ primary facilities. Finally, we examined whether care continuity could contribute to racial disparities by studying racial differences in the availability of nephrologists with high-quality primary facilities and in the quality of patients’ initial dialysis facilities.

## Methods

### Data Sources and Population

The University of Southern California institutional review board approved this study. A waiver of informed consent was provided because this was a secondary analysis of administrative data, and the research posed minimal risk to participants, in accordance with 45 CFR §46. This study followed the Strengthening the Reporting of Observational Studies in Epidemiology (STROBE) reporting guidelines for observational cohort studies.

We used the US Renal Data System (USRDS), a national registry of patients with ESKD,^15^ which includes the CMS-2728 Form, a dataset with demographic and clinical data for all patients initiating dialysis irrespective of insurance^16^; Medicare enrollment; FFS Medicare claims; CMS’s annual dialysis facility survey^17^; and zip codes of patients’ residences and facility addresses. To the USRDS, we linked annual facility quality ratings from DFC (from 1 to 5 stars),^18^ physician specialty from the National Plan and Provider Enumeration System,^19^ and zip code–level sociodemographic information from Census data and the American Community Survey.^20^ See the eAppendix and eTables 1 and 2 in Supplement 1 for technical specifications.

We studied patients aged 18 years and older initiating dialysis for ESKD in the US between January 1, 2015, and October 31, 2020, and followed up patients for 1 year (last day of follow-up was October 31, 2021). Analyses concluded January 26, 2025. Because we identified the patients’ nephrologist with predialysis Medicare claims, we required at least 12 months of FFS Medicare coverage before dialysis. We required that patients’ predialysis nephrologists billed at least 1 dialysis claim in that year (98.8% of total starts).^21^ We excluded patients with preemptive kidney transplants but included patients who received transplants after commencing dialysis. We required that the nephrologist’s primary facility and patients’ first dialysis facility had DFC ratings, were freestanding, and had pediatric patient volumes less than 25% (pediatric-focused facilities were substantially different from other facilities; eTable 3 in Supplement 1).

### Identifying Predialysis Nephrologists and Primary Facilities

We assigned the predialysis nephrologist by determining who billed the plurality of nephrologists’ outpatient evaluation and management claims 180 days before starting dialysis. Patients without a pre-ESKD outpatient nephrologist were assigned the nephrologist billing the last inpatient predialysis evaluation and management claim. Approximately 76% of patients had outpatient nephrologists. We conducted sensitivity analyses on this definition, including omitting patients with only inpatient nephrologists.

The nephrologist’s primary facility for a given year was where they managed the plurality of patient-months in the previous calendar year. On average, 62% of nephrologists’ patient-months were in primary facilities. We used the previous year’s share of patient-months to avoid simultaneity bias with the outcome.

### Outcomes, Exposures, and Covariates

Our first set of outcomes examined whether the starting facility was the nephrologist’s primary facility (hereafter, primary facility starts), high quality (4 or 5 stars) in the year of initiation, and close to the patient. We also examined person-year rates of mortality and hospitalization in dialysis year one. We used a previously described algorithm to identify hospitalizations from USRDS claims.^22^ The exposure time was the observed follow-up time in the first year of dialysis (ie, patients contributed less than a year if they died or were lost to follow-up prior to 1 year).

The exposures were the 5-star rating (a continuous variable) of the predialysis nephrologist’s primary facility in the year prior to dialysis start (to avoid simultaneity bias) and its distance to the patient (close or distant). Distances were calculated as the straight-line distance between the population-weighted centroid of the patient’s residential zip code^23^ and the geocoded street address of the dialysis facility (geocoding was performed using Google Maps^24^). To account for regional variability in population density, we classified a facility close to a patient if its distance was in the bottom quartile of all facilities within a regional network, defined as the set of zip codes of facilities where the nephrologist’s patients initiated dialysis. Conceptually, this regional network contains all facilities available to the patient (see eTables 4-6 in Supplement 1 for regional network characteristics).

Subsequently, we examined racial and ethnic disparities in whether nephrologists’ primary facilities were high quality and whether patients’ starting facilities were high quality. Conceptually, the former investigates whether patients from minoritized racial and ethnic groups have fewer available high-quality facilities, and the latter investigates disparities in outcomes.

We adjusted for patient characteristics (age, sex, self-designated race and ethnicity [Asian, Black, Hispanic, non-Hispanic White, and other, which includes American Indian, Alaska Native, Hawaiian, Pacific Islander, multiracial, any other race, or unknown], dual eligibility, employment status at dialysis start, and comorbidities from historical claims^25^), residential zip code characteristics (population; median income; median rent; proportion of zip code with a high school degree; average work commuting times; and metropolitan, micropolitan, or rural status), and county-week COVID-19 severity for those starting dialysis after the COVID-19 pandemic.^26^ We adjusted for sex, race, and ethnicity because they are associated with disparities in dialysis quality and outcomes.

For the outcomes of primary facility starts, high-quality facility, and facility close to the patient, we also adjusted for the previous year’s market share in the regional network because larger facilities will have more dialysis starts even if starts are random. A facility’s market share from the previous year was the proportion of patient-months in the regional network provided by that facility. When modeling primary facility starts, we adjusted for the primary facility’s market share. When examining high-quality and close facility starts, we adjusted for the market share of all high-quality and all close facilities in the regional network, respectively. We used the previous year’s market share to avoid simultaneity bias from dialysis starts impacting market share.

### Statistical Analysis

We produced descriptive statistics, stratifying patients by the quality and distance of the nephrologist’s preferred facility. We used Pearson χ^2^ test to compare categorical variables and the Kruskal-Wallis test to compare continuous variables. We also graphed the unadjusted probabilities and rates of all 7 aforementioned outcomes. When examining the probability of starting dialysis at the primary facility, nearby facilities, and high-quality facilities, we compared with the market share to demonstrate whether outcomes occurred in excess of what was expected due to random chance.

We subsequently conducted adjusted analyses. For facility characteristic analyses, we used linear regression by regressing each outcome on the quality and distance of the nephrologist’s primary facility, covariates, and Hospital Service Area (HSA)–level^27^ fixed effects (ie, a unique intercept for each HSA that may correlate with the independent variables and the outcome). HSA fixed effects control for unobserved confounders invariant to HSAs and restrict comparisons to patients with different nephrologists living within the same HSA.^28^ We conducted subgroup analyses by interacting the preferred facility’s quality and distance. We used HSA-level cluster-robust SEs. For mortality and hospitalizations, we used Poisson regression with fixed effects, accounting for exposure time. We used robust SEs, which ensures unbiased estimates even in cases of overdispersion.^28^ To ease with interpretation, we applied incident rate ratios to patient-year hospitalization and mortality rates to estimate the absolute change in rates.

For the outcomes of racial and ethnic disparities in whether nephrologists’ primary facilities were high quality and whether patients’ starting facilities were high quality, using linear regression, we regressed the quality of nephrologists’ primary facilities and patients’ starting facilities on race and ethnicity and dual eligibility (enrollment in both Medicare and Medicaid). We further tested whether disparities remained within the same HSA by incorporating HSA fixed effects. In these analyses, we did not adjust for market share because doing so masks disparities in access, although we included market share in sensitivity analysis.

We conducted 6 categories of sensitivity checks: (1) varied the assignment criteria for the predialysis nephrologist (eg, excluded patients without an outpatient nephrologist); (2) varied the level of geographic fixed effects (eg, used hospital referral region instead of HSA); (3) restricted to specific subgroups (eg, those starting dialysis before the COVID-19 pandemic, starting with in-center hemodialysis, those in metropolitan areas, and those with 2 or more close facilities); (4) omitted market share adjustments from the dialysis start outcomes and included market share adjustments in the disparities outcomes; (5) for mortality and hospitalization outcomes, we tested various exposure periods (eg, requiring patients have at least 30 or 90 days of dialysis); and (6) tested different definitions of facility distance. We used 2-tailed tests with an α of .05, adjusting *P* values of primary analyses using an adaptive correction that preserves the false discovery rate with correlated outcomes.^29^ We did not adjust *P* values for subgroup analyses, given the number performed and their ad hoc nature. We used SAS statistical software version 9.4 (SAS Institute) and Stata statistical software version 14.0 MP edition (StataCorp) for statistical analyses.

## Results

### Analytic Sample

Of 143 776 patients (median [IQR] age, 73 [67-79] years; 64 447 female [45%]; 4989 Asian [3%]; 28 515 Black [20%]; 11 296 Hispanic [8%]; 96 639 non-Hispanic White [67%]; 2337 other racial and ethnic categories [2%]), 68 704 (40%) lived in zip codes with median income below $50 000 (Table 1 and eTable 7 and the eFigure in Supplement 1). Thirty-one percent of patients (44 978 patients) had predialysis nephrologists with nearby primary facilities and 45% (64 186 patients) with high-quality primary facilities. The 9140 nephrologists (36 662 nephrologist-years) in our sample had 5161 primary facilities out of 6441 freestanding, nonpediatric facilities.

**Table 1.** Patient Characteristics, by Predialysis Nephrologists’ Primary Facilities^a^

Characteristic	Patients, No. (%)						
	Distance from referring nephrologists’ primary facility		*P* value	Quality of referring nephrologists’ primary facility			*P* value^b^
	Close (n = 44 978)	Far (n = 98 798)		Low, 1-2 stars (n = 22 539)	Average, 3 stars, (n = 57 051)	High, 4-5 stars, (n = 64 186)	
Age, mean (SD), y	72.2 (10.8)	71.8 (10.9)	<.001	71.5 (11.1)	71.8 (10.9)	72.2 (10.7)	<.001
Sex							
Male	24 772 (55.1)	54 557 (55.2)	.61	12 155 (53.9)	31 344 (54.9)	35 830 (55.8)	<.001
Female	20 206 (44.9)	44 241 (44.8)		10 384 (46.1)	25 707 (45.1)	28 356 (44.2)	
Race and ethnicity							
Asian	1480 (3.3)	3509 (3.6)	<.001	513 (2.3)	1702 (3.0)	2774 (4.3)	<.001
Black	8257 (18.4)	20 258 (20.5)		5638 (25.0)	12 237 (21.4)	10 640 (16.6)	
Hispanic	3331 (7.4)	7965 (8.1)		1414 (6.3)	4173 (7.3)	5709 (8.9)	
White, non-Hispanic	31 200 (69.4)	65 439 (66.2)		14 763 (65.5)	38 149 (66.9)	43 727 (68.1)	
Other^c^	710 (1.6)	1627 (1.6)		211 (0.9)	790 (1.4)	1336 (2.1)	
Dual eligible	13 153 (29.2)	29 783 (30.1)	<.001	7050 (31.3)	16 929 (29.7)	18 957 (29.5)	<.001
Herfindahl-Hirschman Index of regional network, mean (SD)^d^	5460 (2227)	5218 (2057)	<.001	4976 (2067)	5224 (2078)	5468 (2147)	<.001
Comorbid conditions							
Diabetes	32 851 (73.0)	72 028 (72.9)	.60	16 426 (72.9)	41 623 (73.0)	46 830 (73.0)	.97
Congestive heart failure	30 575 (68.0)	67 264 (68.1)	.69	15 711 (69.7)	38 916 (68.2)	43 212 (67.3)	<.001
Hyperlipidemia	32 580 (72.4)	71 581 (72.5)	.95	14 287 (63.4)	41 329 (72.4)	48 545 (75.6)	<.001
Hypertension	43 117 (95.9)	94 849 (96.0)	.21	21 010 (93.2)	54 619 (95.7)	62 337 (97.1)	<.001
Ischemic heart disease	29 098 (64.7)	64 162 (64.9)	.36	15 026 (66.7)	37 094 (65.0)	41 140 (64.1)	<.001
Atrial fibrillation	14 266 (31.7)	31 129 (31.5)	.43	7155 (31.7)	18 067 (31.7)	20 173 (31.4)	.56
Acute myocardial infarction	4498 (10.0)	10 236 (10.4)	.04	2533 (11.2)	5970 (10.5)	6231 (9.7)	<.001
Chronic obstructive pulmonary disease	15 283 (34.0)	33 375 (33.8)	.46	8117 (36.0)	19 750 (34.6)	20 791 (32.4)	<.001
Depression	13 932 (31.0)	30 956 (31.3)	.18	7100 (31.5)	17 641 (30.9)	20 147 (31.4)	.13
Stroke or transient ischemic attack	5279 (11.7)	12 136 (12.3)	<.01	2866 (12.7)	7094 (12.4)	7455 (11.6)	<.001
Population density of residence							
Rural	3135 (7.0)	8447 (8.5)	<.001	2029 (9.0)	4581 (8.0)	4972 (7.7)	<.001
Metropolitan	35 314 (78.5)	79 443 (80.4)		18 049 (80.1)	45 725 (80.1)	50 983 (79.4)	
Micropolitan	6529 (14.5)	10 908 (11.0)		2461 (10.9)	6745 (11.8)	8231 (12.8)	
Residential zip code characteristics							
Population size, mean (SD)	27 615 (18 600)	29 043 (19 733)	<.001	27 693 (19 347)	28 445 (19 134)	29 048 (19 633)	<.001
Median income, mean (SD), $	58 678 (25 110)	58 553 (24 142)	.06	52 750 (21 619)	57 105 (23 112)	61 965 (25 976)	<.001
Population below poverty line, mean (SD), %	15.2 (8.7)	15.3 (8.9)	.52	17.0 (9.4)	15.6 (8.8)	14.4 (8.5)	<.001
Population unemployed, mean (SD), %	7.2 (3.9)	7.3 (3.9)	<.001	8.4 (4.5)	7.4 (3.9)	6.7 (3.6)	<.001
Population without high school diploma, mean (SD), %	13.5 (8.7)	13.7 (8.9)	<.001	14.4 (8.5)	13.7 (8.7)	13.3 (9.0)	<.001
Median rent, mean (SD), $	990 (376)	998 (366)	<.001	914 (318)	972 (350)	1044 (395)	<.001
Population with travel time to work of zip code, mean (SD), %							
<10 min	14.4 (8.9)	13.5 (8.8)	<.001	13.6 (8.6)	13.7 (8.7)	14.0 (9.0)	<.001
10-19 min	31.0 (11.0)	29.3 (10.2)	<.001	29.9 (10.6)	29.9 (10.4)	29.7 (10.5)	<.001
20-29 min	19.9 (7.1)	20.4 (7.0)	<.001	20.6 (7.1)	20.4 (7.0)	20.0 (7.1)	<.001
30-59 min	26.2 (11.5)	27.8 (11.0)	<.001	27.5 (11.3)	27.4 (11.2)	27.2 (11.2)	.06
≥60	8.5 (6.4)	9.0 (6.7)	<.001	8.4 (6.2)	8.7 (6.5)	9.1 (6.8)	<.001
COVID-19 severity^e^							
Weekly county COVID-19 rate, cases/100 000 people	25.9 (63.9)	27.3 (71.7)	.90	29.9 (67.5)	27.7 (71.8)	26.0 (68.1)	<.001
Weekly county COVID-19 mortality, deaths/100 000 people	0.68 (2.5)	0.71 (2.6)	.02	0.8 (2.2)	0.7 (2.7)	0.7 (2.6)	<.001

^a^ Data are from the US Renal Data System data. This table is truncated for brevity. See eTable 7 in Supplement 1 for a full set of characteristics.

^b^ *P* values were computed using the Pearson χ^2^ test for categorical variables and the Kruskal-Wallis test for continuous variables.

^c^ Other race and ethnicity refers to American Indian, Alaska Native, Hawaiian, Pacific Islander, multiracial, any other race, or unknown.

^d^ The Herfindahl-Hirschman Index is a measure of market consolidation and ranges from 0 to 10 000, where 10 000 means the region is a true monopoly.

^e^ COVID-19 severity was estimated only for the 40 771 patients who initiated dialysis on or after the onset of the COVID-19 pandemic.

### Dialysis Starts

**Figure 1.**
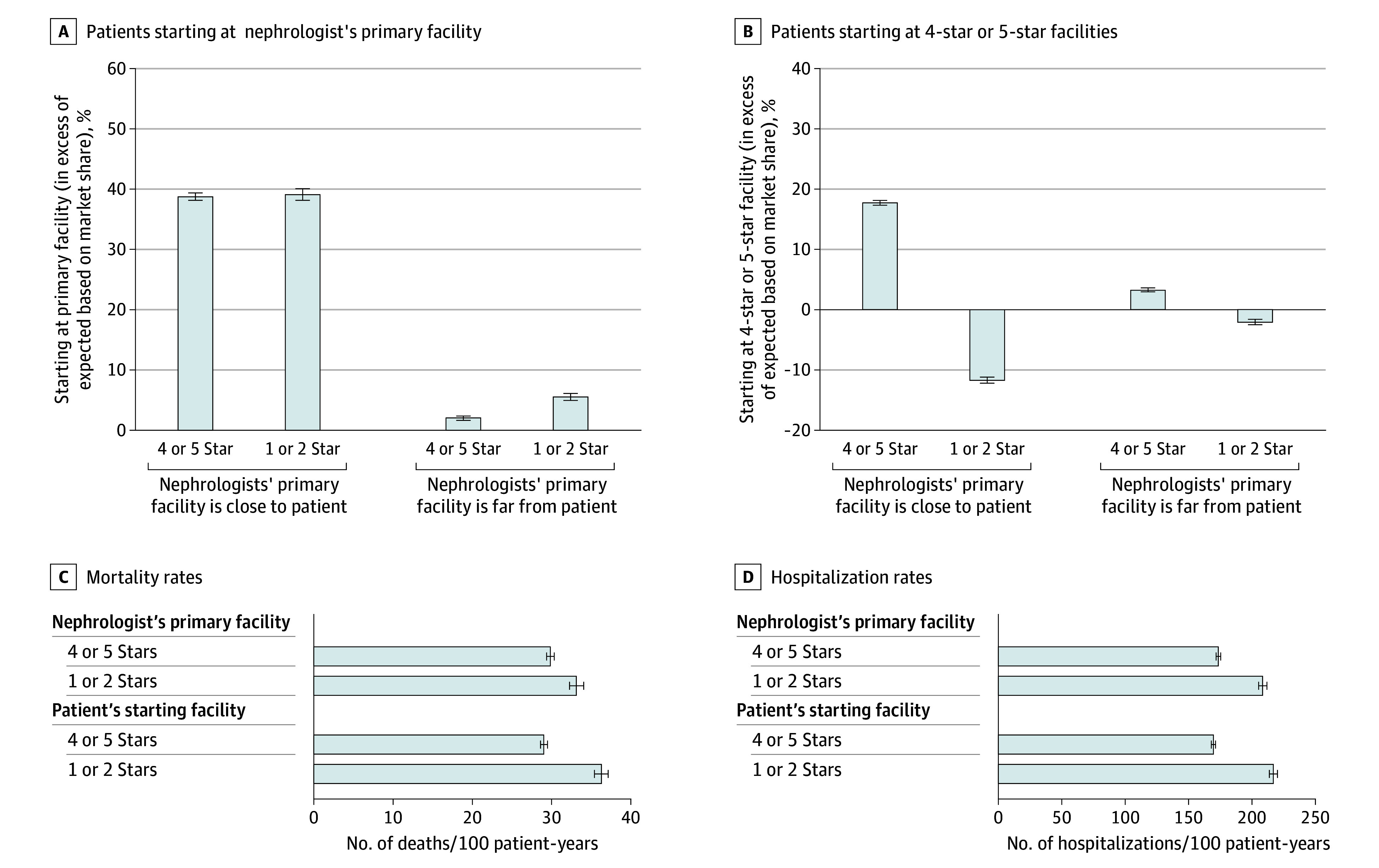
Outcomes Stratified by Characteristics of the Predialysis Nephrologist’s Primary Facility and the Patient’s First Facility, Among Fee-for-Service Medicare Patients Initiating Dialysis, 2015-2020 A, Proportion of patients starting at the nephrologist’s primary facility (where they managed the plurality of dialysis patients in the previous year). B, Patients starting at 4-star or 5-star (high-quality) facilities. Panels A and B depict starts in excess of market share (ie, the difference between the proportion of starts and the market share in the patient’s region, which is what one would expect if starts were random) stratified by characteristics of the predialysis nephrologist’s primary facility. Panels C and D show mortality and hospitalization rates per 100 person-years, respectively; 95% CIs (error bars) are shown for all statistics. Data are from the US Renal Data System (eTables 8 and 9 in Supplement 1).

In unadjusted analysis, we quantified whether the probability of starting dialysis at a primary, close, or high-quality facility exceeded what was expected by market share. When primary facilities were close, primary facility starts exceeded market share by 38 percentage points (pp) (64%, despite having only 26% market share) (Figure 1A; eTable 8 in Supplement 1). Thus, when primary facilities were high quality and close, high-quality starts were 18 pp over the market share of all high-quality facilities in the region; when low-quality and close, high-quality starts were 12 pp below market share (Figure 1B). Primary facility starts and high-quality starts exceeded market share by less than 6 pp when primary facilities were distant.

Findings were similar on adjusted analysis (Table 2). Primary facility starts were slightly lower as the primary facility’s quality increased (0.5 pp lower for every 1-star increase in rating; 95% CI, 0.1-0.8 pp; adjusted *P* = .03). For patients whose nephrologists had nearby primary facilities, we observed no material difference between primary facility starts and quality—a 0.9 pp increase for each additional star in rating. (We do not report significance for subgroup analyses.) Conversely, primary facility starts were 33.9 pp (95% CI, 33.0-34.9 pp; adjusted *P* < .001) more likely when the primary facility was nearby vs distant. Patients were 20.2 pp (95% CI, 19.2-21.2 pp; adjusted *P* < .001) more likely to start dialysis in nearby facilities when the primary facility was close vs distant and 7.4 pp (95% CI, 6.9-7.9 pp; adjusted *P* < .001) more likely to have a high-quality start for each additional star in rating.

**Table 2.** Outcomes Associated With Characteristics of the Predialysis Nephrologist’s Primary Facility, Among Fee-for-Service Medicare Patients Initiating Dialysis, 2015-2020

Characteristic	Change in referral outcomes, percentage points (95% CI)^a^			Absolute rate change (95% CI)^b^	
	Starting at primary facility	Starting at close facility	Starting at 4- or 5-star facility	No. of deaths per 100 person-years	No. of hospitalizations per 100 person-years
Primary analysis					
Far from patient	0 [Reference]	0 [Reference]	0 [Reference]	0 [Reference]	0 [Reference]
Close to patient^c^	33.9 (33.0 to 34.9)^d^	20.2 (19.2 to 21.2)^d^	−0.3 (−0.9 to 0.2)	1.33 (0.56 to 2.11)^e^	−2.63 (−5.39 to 0.17)
For every 1-star increase in primary facility’s 5-star rating^f^	−0.5 (−0.8 to −0.1)^g^	0.4 (0.1 to 0.8)^g^	7.4 (6.9 to 7.9)^d^	−0.40 (−0.85 to 0.05)	−4.48 (−6.12 to −2.82)^d^
Change when primary facility goes from 1 to 5 stars^h^	−1.9 (−3.4 to −0.4)^g^	1.6 (0.3 to 3.0)^g^	29.6 (27.6 to 31.6)^d^	−1.58 (−3.28 to 0.22)	−17.36 (−23.46 to −11.07)^d^
Subgroup analysis No. 1: when nephrologist’s primary facility is close to patient					
For every 1-star increase in primary facility’s 5-star rating	0.9 (0.3 to 1.5)	−0.7 (−1.3 to −0.1)	13.6 (12.9 to 14.3)	−0.79 (−1.52 to −0.04)	−5.86 (−8.50 to −3.19)
Change when primary facility goes from 1 to 5 stars	3.5 (1.0 to 6.1)	−2.9 (−5.3 to −0.5)	54.4 (51.6 to 57.1)	−3.05 (−5.71 to −0.17)	−22.49 (−31.99 to −12.49)
Subgroup analysis No. 2: when nephrologist’s primary facility is far from patient					
For every 1-star increase in primary facility’s 5-star rating	−1.0 (−1.4 to −0.6)	0.9 (0.4 to 1.3)	5.0 (4.5 to 5.5)	−0.24 (−0.76 to 0.28)	−3.93 (−5.80 to −2.05)
Change when primary facility goes from 1 to 5 stars	−4.0 (−5.6 to −2.5)	3.4 (1.7 to 5.1)	20.0 (18.0 to 22.0)	−0.96 (−2.93 to 1.13)	−15.31 (−22.29 to −8.09)
Subgroup analysis No. 3: when nephrologist’s primary facility is 5 stars					
Far from patient	0 [Reference]	0 [Reference]	0 [Reference]	0 [Reference]	0 [Reference]
Close to patient	36.9 (35.6 to 38.2)	17.7 (16.2 to 19.2)	13.2 (12.1 to 14.3)	0.51 (−0.84 to 1.93)	−5.09 (−9.56 to −0.50)
Subgroup analysis No. 4: when nephrologist’s primary facility is 1 star					
Far from patient	0 [Reference]	0 [Reference]	0 [Reference]	0 [Reference]	0 [Reference]
Close to patient	29.3 (27.4 to 31.3)	24.0 (21.9 to 26.1)	−21.3 (−22.8 to −19.7)	2.86 (0.64 to 5.23)	2.08 (−5.94 to 10.40)

^a^ Percentage point changes were estimated using multivariable regression at the patient level, adjusted for patient demographics, comorbid conditions, characteristics of zip code of residence, and Hospital Service Area–level fixed effects. Referral outcomes were adjusted for what is expected on the basis of facility market share and estimated with linear regression.

^b^ Mortality and hospitalizations were estimated using Poisson regression. Per-person rates estimated by applying incident rate ratios to baseline rates. *P* values were corrected for multiple hypothesis testing assuming a false discovery rate of 5% (*P* value adjustments were not performed for subgroup analyses).

^c^ Close facilities are those that are in the bottom quartile of distance to the patient among the facilities in the nephrologist’s referral region.

^d^ Adjusted *P* < .001.

^e^ Adjusted *P* < .01.

^f^ Shown is the marginal effect of increasing the star-rating of the primary facility by 4 stars.

^g^ Adjusted *P* < .05.

^h^ Five-star ratings of the primary facility were modeled as a continuous variable.

In unadjusted analysis, patients whose nephrologists’ primary facilities were high quality had lower mortality and hospitalization rates than patients whose nephrologists’ primary facilities were low quality (Figures 1C and 1D; eTable 9 in Supplement 1). After adjusting for confounders, mortality differences were not significant by primary facility quality, but differences in hospitalization persisted. For each additional star in primary facility rating, patients had a 4.5 per 100 person-year lower (95% CI, 2.8-6.1 hospitalizations per 100 person-years; adjusted *P* < .001) hospitalization rate, or a 17.4 per 100 person-year (95% CI, 11.1-23.5 hospitalizations per 100 person-years) decrease in hospitalization rates when the primary facility had 5 stars vs 1 star (Table 2).

### Differences by Race and Ethnicity and Dual Eligibility

**Figure 2.**
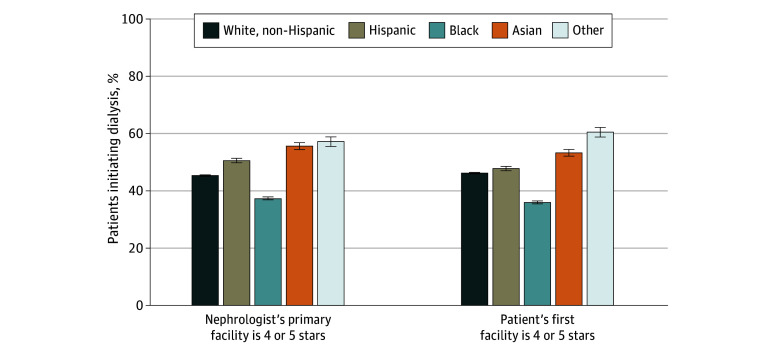
Quality of the Predialysis Nephrologist’s Primary Facility and the Patient’s Starting Facility by Race and Ethnicity, Among Fee-for-Service Medicare Patients Initiating Dialysis, 2015-2020 Other race refers to American Indian, Alaska Native, Hawaiian, Pacific Islander, multiracial, any other race, or unknown. Data are from the US Renal Data System (eTable 10 in Supplement 1).

In unadjusted analysis, Black patients were least likely to have nephrologists whose primary facility was high quality (37.3% [95% CI, 36.8%-37.8%] vs 45.2% [95% CI, 45.0%-45.5%] among non-Hispanic White patients) or to start dialysis in high-quality facilities (36.0% [95% CI, 35.5%-36.5%] vs 46.2% [95% CI, 45.9%-46.5%] among non-Hispanic White patients) (Figure 2; eTable 10 in Supplement 1). In adjusted analysis, compared with non-Hispanic White patients, Black patients were 4.6 pp (95% CI, 3.2-6.0 pp) less likely to have nephrologists with high-quality primary facilities (Table 3) and were 5.8 pp (95% CI, 4.3-7.2 pp) less likely to start in high-quality facilities. For patients living within the same HSA, compared with non-Hispanic White patients, Black patients were 2.0 pp (95% CI, 1.0-3.0 pp; adjusted *P* < .001) less likely to have nephrologists preferring high-quality facilities and 2.8 pp (95% CI, 1.7-3.9 pp; adjusted *P* < .001) less likely to start dialysis in high-quality facilities. We did not observe significant differences among other racial or ethnic groups or by dual eligibility. The findings did not materially change on sensitivity analyses (eTables 11-17 in Supplement 1).

**Table 3.** Disparities in the Quality of the Nephrologist’s Primary Facility and the Quality of the Patient’s First Dialysis Facility, by Race, Ethnicity, and Dual Eligibility, Among Fee-for-service Medicare Patients Initiating Dialysis, 2015-2020

Patient characteristic	Change, percentage points (95% CI)^a^	
	Overall	Within the same HSA
Change in likelihood that the nephrologist’s primary facility is 4 or 5 stars		
Race and ethnicity		
Asian	6.0 (2.8 to 9.2)	2.3 (−0.2 to 4.9)
Black	−4.6 (−6.0 to −3.2)	−2.0 (−3.0 to −1.0)^b^
Hispanic	4.1 (1.1 to 7.1)	0.0 (−1.3 to 1.4)
White, non-Hispanic	0 [Reference]	0 [Reference]
Other^c^	9.6 (6.0 to 13.1)	1.0 (−1.4 to 3.3)
Dual eligibility^d^		
Nondual	0 [Reference]	0 [Reference]
Dual	0.4 (−0.5 to 1.4)	−0.4 (−1.1 to 0.3)
Change in likelihood of initiating dialysis at a 4-star or 5-star facility		
Race and ethnicity		
Asian	5.2 (1.8 to 8.7)	1.8 (−0.5 to 4.0)
Black	−5.8 (−7.2 to −4.3)	−2.8 (−3.9 to −1.7)^b^
Hispanic	2.3 (−0.8 to 5.5)	−0.7 (−2.1 to 0.7)
White, non-Hispanic	0 [Reference]	0 [Reference]
Other^c^	11.2 (7.7 to 14.7)	1.9 (−0.9 to 4.6)
Dual eligibility^d^		
Nondual	0 [Reference]	0 [Reference]
Dual	0.6 (−0.3 to 1.6)	0.1 (−0.5 to 0.8)

Abbreviation: HSA, hospital service area.

^a^ Percentage point changes were estimated using linear regression at the patient level, adjusted for patient demographics, comorbid conditions, characteristics of zip code of residence, with and without HSA-level fixed effects. *P* values were corrected for multiple hypothesis testing assuming a false discovery rate of 5% (*P* values adjustments only performed for within-HSA analyses).

^b^ Adjusted *P* < .001.

^c^ Other race and ethnicity refers to American Indian, Alaska Native, Hawaiian, Pacific Islander, multiracial, any other race, or unknown.

^d^ Dual eligible means simultaneous enrollment in Medicare and Medicaid.

## Discussion

In this cohort study, primary facility starts were common when nephrologists’ primary facilities were close to patients, even when the facilities were low quality. Patients whose nephrologists had low-quality primary facilities—and by extension low-quality starts—had higher mortality and hospitalizations. Black patients were more likely to have predialysis nephrologists with low-quality primary facilities and to have low-quality starts, disparities that persisted even when comparing patients within the same HSA.

Although we could not observe patients’ motivations behind these decisions, our findings suggest that patients prioritize care continuity and proximity over quality when choosing dialysis facilities. Few studies have examined care continuity in dialysis,^9,10^ and, to our knowledge, none have studied care continuity when initiating dialysis. Patients in primary care settings prefer preserving care continuity,^30,31^ which is associated with reduced mortality and hospitalizations.^6,32,33^ Our study had mixed findings: care continuity with nephrologists with low-quality primary facilities was associated with worse outcomes.

Five-star ratings aim to help patients identify high-quality and low-quality dialysis facilities,^18^ but they did not appear to help patients avoid low-quality primary facilities. We offer some hypotheses. First, patients may be unaware of 5-star ratings. Alternatively, quality may not be a high priority for patients. Finally, high-quality facilities may be inaccessible to patients whose nephrologists have low-quality primary facilities; for instance, nephrologists with low-quality primary facilities may only have a network of lower quality facilities. Nevertheless, these findings suggest that 5-star ratings may be less influential than intended and complement other data demonstrating that the Quality Incentive Program,^34^ a companion CMS dialysis quality program, did not change facility behavior.^35^

A less charitable explanation for primary facility starts is that nephrologists might act on financial conflicts of interest by self-referring patients to low-quality facilities where they own equity or have medical directorships.^36,37^ Although a previous study^38^ showed that nephrologist ownership of dialysis facilities might not result in poor outcomes and could improve home dialysis rates, it did not examine patients’ selection of dialysis facilities. Future work should examine whether and to what extent nephrologists can help patients select high-quality facilities.

Although racial and ethnic disparities in dialysis quality are well known, mechanisms are not as well articulated. Our study suggests that one potential mechanism by which disparities manifest is through care continuity because Black patients were more likely treated by nephrologists with low-quality primary facilities. Structural disparities in the availability of high-quality primary facilities mirror other disparities that perpetuate poor health care outcomes among Black patients, especially in kidney care.^39,40,41^ These disparities are made more salient by the overrepresentation of Black patients in the ESKD population.^15,42^

CMS has prioritized addressing disparities in ESKD and adjusts for dual Medicare-Medicaid enrollment in its End-Stage Renal Disease Treatment Choices model.^43^ Our findings dovetail with concerns that dialysis-centric quality programs may disadvantage safety-net facilities without benefiting patients,^44,45^ particularly because these policies do not address the transition to dialysis. The Kidney Care Choices model—and value-based care more broadly—aims to smooth the transition to care by holding nephrologists accountable for better multidisciplinary care upstream.^46,47,48^ However, improved care coordination would still not address the uneven distribution of high-quality facilities available to patients. Alternative polices such as incentives that attract high-quality nephrologists and dialysis facilities to safety-net settings may be required to alleviate this disparity.

### Limitations

Our study has limitations. First, our findings are limited by residual confounding and an inability to observe some clinical details, such as the managing nephrologist. To mitigate residual confounding, we incorporated HSA fixed effects to adjust for unobserved, HSA-invariant factors, although selection bias at a more granular level may still remain. We also tested alternate assignments of the nephrologist. Second, we could not directly observe travel distances because we lacked patients’ street addresses. However, this measurement error should bias our results to null, and we tested alternate measures of distance. Third, facility 5-star ratings may not be a suitable proxy for facility quality. However, our findings are relevant to evaluating the ratings’ policy purpose: to help patients choose high-quality facilities. Moreover, our study examined specific clinical outcomes, not just quality indicators. Fourth, although FFS Medicare is the plurality payer for ESKD, our results may not generalize beyond FFS Medicare.^49^

## Conclusions

In this cohort study of FFS Medicare patients who initiated dialysis for ESKD, primary facility starts were common, especially if primary facilities were close to patients and even when they were low quality. Given that starts did not correlate with 5-star ratings, policymakers may wish to bolster the DFC’s effectiveness, including improved publicity to patients. Primary facility starts may exacerbate racial disparities in dialysis quality because nephrologists with high-quality facilities were less accessible to Black patients. Supply-side policies that promote improved access to high-quality dialysis facilities, such as subsidies or incentives to move, may be necessary to alleviate these disparities.

## Notes

Supplement 1. **eAppendix**. Technical Specifications **eTable 1.** Evaluation and Management Codes **eTable 2.** Monthly Capitated Payment Dialysis Codes **eFigure.** CONSORT Diagram **eTable 3.** Mean Characteristics of Pediatric versus Non-Pediatric Facilities **eTable 4.** Mean Characteristics of Regional Networks **eTable 5.** Number of Close and 4- or 5-Star Facilities within Patients’ Regional Networks **eTable 6.** The 20th, 25th, and 30th Percentile Distances (in kilometers) between Patients and Dialysis Facilities in their Regional Network, Stratified by Population Density **eTable 7.** Patient Comorbid Conditions, Stratified by Their Pre-Dialysis Nephrologists’ Primary Facilities **eTable 8.** Unadjusted Probabilities of Starting at the Nephrologist’s Primary Facility and of Starting at a 4- or 5-Star Facility **eTable 9.** Unadjusted Person-Year Rates of Mortality and Hospitalization, Stratified by the Quality of the Nephrologist’s Primary Facility and the Starting Facility **eTable 10.** Unadjusted Probability of Having a Nephrologist with a High-Quality Primary Facility and of Starting at a High-Quality Facility, Stratified by Race **eTable 11.** Sensitivity Analysis of Starting Facility Outcomes, Comparing Nephrologists’ Primary Facilities that are Nearby to Primary Facilities that are Far from Patient **eTable 12.** Sensitivity Analysis of Starting Facility Outcomes, Comparing Nephrologists’ Primary Facilities that are High Quality to Low Quality **eTable 13.** Sensitivity Analysis of Mortality and Hospitalization Outcomes, Comparing Nephrologists’ Primary Facilities that are Nearby to Primary Facilities that are Far from Patient **eTable 14.** Sensitivity Analysis of Mortality and Hospitalization Outcomes, Comparing Primary Facilities that are High Quality to Low Quality **eTable 15.** Sensitivity Analysis of Starting Facility Outcomes, Comparing Primary Facilities that are Close to Facilities that are Far from the Patient, Using Different Definitions of Distance **eTable 16.** Sensitivity Analysis of Starting Facility Outcomes, Comparing Primary Facilities that are High Quality to Facilities that are Low Quality, Using Different Definitions of Distance **eTable 17.** Sensitivity Analysis of Disparities between Black and White Patients
eReferences

Supplement 2.
Data Sharing Statement
