# Supplementary material for: Care Continuity, Nephrologists’ Dialysis Facility Preferences, and Outcomes
Source: JAMA Health Forum. 2025 Apr 11;6(4):e250423. doi: 10.1001/jamahealthforum.2025.0423 (PMC11992609; doi:10.1001/jamahealthforum.2025.0423)
Supplement: Supplement 1. — eAppendix. Technical Specifications eTable 1. Evaluation and Management Codes eTable 2. Monthly Capitated Payment Dialysis Codes eFigure. CONSORT Diagram eTable 3. Mean Characteristics of Pediatric versus Non-Pediatric Facilities eTable 4. Mean Characteristics of Regional Networks eTable 5. Number of Close and 4- or 5-Star Facilities within Patients’ Regional Networks eTable 6. The 20th, 25th, and 30th Percentile Distances (in kilometers) between Patients and Dialysis Facilities in their Regional Network, Stratified by Population Density eTable 7. Patient Comorbid Conditions, Stratified by Their Pre-Dialysis Nephrologists’ Primary Facilities eTable 8. Unadjusted Probabilities of Starting at the Nephrologist’s Primary Facility and of Starting at a 4- or 5-Star Facility eTable 9. Unadjusted Person-Year Rates of Mortality and Hospitalization, Stratified by the Quality of the Nephrologist’s Primary Facility and the Starting Facility eTable 10. Unadjusted Probability of Having a Nephrologist with a High-Quality Primary Facility and of Starting at a High-Quality Facility, Stratified by Race eTable 11. Sensitivity Analysis of Starting Facility Outcomes, Comparing Nephrologists’ Primary Facilities that are Nearby to Primary Facilities that are Far from Patient eTable 12. Sensitivity Analysis of Starting Facility Outcomes, Comparing Nephrologists’ Primary Facilities that are High Quality to Low Quality eTable 13. Sensitivity Analysis of Mortality and Hospitalization Outcomes, Comparing Nephrologists’ Primary Facilities that are Nearby to Primary Facilities that are Far from Patient eTable 14. Sensitivity Analysis of Mortality and Hospitalization Outcomes, Comparing Primary Facilities that are High Quality to Low Quality eTable 15. Sensitivity Analysis of Starting Facility Outcomes, Comparing Primary Facilities that are Close to Facilities that are Far from the Patient, Using Different Definitions of Distance eTable 16. Sensitivity Analysis of Starting Facility Outcomes, Comp [file jamahealthforum-e250423-s001.pdf]

Supplemental Online Content

Lin E, Lung KI, Rapista D, et al. Care continuity, nephrologists' dialysis facility preferences, and outcomes. *JAMA Health Forum*. 2025;6(4):e250423. doi:10.1001/jamahealthforum.2025.0423

eAppendix. Technical Specifications
eTable 1. Evaluation and Management Codes
eTable 2. Monthly Capitated Payment Dialysis Codes
eFigure. CONSORT Diagram
eTable 3. Mean Characteristics of Pediatric versus Non-Pediatric Facilities
eTable 4. Mean Characteristics of Regional Networks
eTable 5. Number of Close and 4- or 5-Star Facilities within Patients' Regional Networks
eTable 6. The 20th, 25th, and 30th Percentile Distances (in kilometers) between Patients and Dialysis Facilities in their Regional Network, Stratified by Population Density
eTable 7. Patient Comorbid Conditions, Stratified by Their Pre-Dialysis Nephrologists' Primary Facilities
eTable 8. Unadjusted Probabilities of Starting at the Nephrologist's Primary Facility and of Starting at a 4- or 5-Star Facility
eTable 9. Unadjusted Person-Year Rates of Mortality and Hospitalization, Stratified by the Quality of the Nephrologist's Primary Facility and the Starting Facility
eTable 10. Unadjusted Probability of Having a Nephrologist with a High-Quality Primary Facility and of Starting at a High-Quality Facility, Stratified by Race
eTable 11. Sensitivity Analysis of Starting Facility Outcomes, Comparing Nephrologists' Primary Facilities that are Nearby to Primary Facilities that are Far from Patient
eTable 12. Sensitivity Analysis of Starting Facility Outcomes, Comparing Nephrologists' Primary Facilities that are High Quality to Low Quality
eTable 13. Sensitivity Analysis of Mortality and Hospitalization Outcomes, Comparing Nephrologists' Primary Facilities that are Nearby to Primary Facilities that are Far from Patient
eTable 14. Sensitivity Analysis of Mortality and Hospitalization Outcomes, Comparing Primary Facilities that are High Quality to Low Quality
eTable 15. Sensitivity Analysis of Starting Facility Outcomes, Comparing Primary Facilities that are Close to Facilities that are Far from the Patient, Using Different Definitions of Distance
eTable 16. Sensitivity Analysis of Starting Facility Outcomes, Comparing Primary Facilities that are High Quality to Facilities that are Low Quality, Using Different Definitions of Distance
eTable 17. Sensitivity Analysis of Disparities between Black and White Patients
eReferences

This supplemental material has been provided by the authors to give readers additional information about their work.

© 2025 Lin E et al. *JAMA Health Forum*.

eAppendix. Technical Specifications

Data Sources

We used the United States Renal Data System (USRDS), a national registry of patients with end-stage kidney disease (ESKD) linked to Medicare Parts A and B claims for patients with fee-for-service (FFS) Medicare.[1] The dataset includes the CMS-2728 Form, which contains demographic and clinical data at the start of dialysis and is submitted for all patients within 45 days of initiating dialysis irrespective of insurance;[2] the Medicare enrollment database, which identifies patients' primary payer; CROWNWeb data, which provides longitudinal clinical details of all patients receiving outpatient dialysis for ESKD in the US; the annual facility survey, which contains dialysis facility-level information;[3] and the zip codes of patients' residences and facilities. We also used publicly available data, which include publicly available facility quality ratings from Medicare's Dialysis Facility Compare (DFC) website;[4] physician specialty information from the National Plan and Provider Enumeration System (NPPES);[5] zip code level sociodemographic information from the American Community Survey (ACS);[6] Area Health Resource Files (AHRF) from the Health Resource and Services Administration to classify zip codes as metropolitan, micropolitan, and rural areas;[7] and the Dartmouth Atlas to map zip codes to Hospital Service Areas (HSA).[8]

Population

We studied patients 18 years and older initiating dialysis (home hemodialysis, in-center hemodialysis, or peritoneal dialysis) for ESKD in the US between January 1, 2015, to October 31, 2020, and we followed patients for one year, with the last day of follow-up October 31, 2021. We required that patients have FFS Medicare coverage at least twelve months prior to starting dialysis, at the time of dialysis, and for at least one year after starting dialysis. We ended our sample at October 31, 2021 because visual inspection of FFS Medicare claims from the USRDS dataset demonstrated missing final action claims in November and December 2021. (December 2021 is the last available month of FFS Medicare claims available to us at the time of the analysis.)

We required that each patient had a pre-dialysis nephrologist with an identified primary facility (defined below). Additionally, patients' first dialysis facility and the managing nephrologists' primary facility must both be freestanding, <25% of patients were pediatric, distance to patients' zip code could be calculated, and a star rating for the facility was available from the DFC program. We chose 25% as a threshold for defining a "pediatric facility" because those facilities were substantially different from other freestanding facilities. (See **eTable 3** for characteristics of pediatric facilities.)

For each patient, we identified the age, sex, race, ethnicity, and date of first outpatient dialysis treatment using the CMS-2728 Form. We determined the starting facility (i.e., the facility of the first outpatient dialysis treatment) using the detailed treatment history dataset, which is derived from CROWNWeb and 72x FFS Medicare outpatient dialysis claims. We determined dual eligibility (concurrent Medicare and Medicaid coverage) using the enrollment dataset. We used one year of inpatient and outpatient FFS Medicare claims prior to starting dialysis to determine if patients had existing comorbidities using the 27 conditions that are a part of the Center for Medicare/Medicaid Service's Chronic Condition Warehouse.[9]

Zip Code and County Characteristics

Characteristics of patients' zip code and county of residence were also identified. From the ACS, we obtained the zip code's population size, median income, proportion below poverty line,

© 2025 Lin E et al. *JAMA Health Forum*.

unemployment rate, median rent, and proportion without a high school degree. We also obtained average commuting times to work from the dataset. The AHRF dataset was used to determine whether the patients' county of residence was metropolitan, micropolitan, or rural. Furthermore, the HSA was identified using patients' zip codes.

Identifying Pre-Dialysis Nephrologists, Nephrologists' Primary Facilities, and Regional Networks

Outpatient dialysis claims do not explicitly identify the nephrologist managing patients' pre-ESKD care. Thus, we developed an algorithm to identify the pre-dialysis nephrologist. First, we identified all nephrologists from the NPPES and their corresponding National Provider Identifiers (NPIs). Next, for each patient, we identified all outpatient evaluation and management (E&M) claims billed by eligible nephrologists in the six months prior to starting dialysis (**eTable 1**). We assigned the pre-dialysis nephrologist as the nephrologist responsible for the plurality of outpatient E&M claims. We broke ties by identifying the plurality nephrologist who billed the last outpatient E&M claim prior to starting dialysis.

For patients without pre-ESKD outpatient nephrology claims, we defined the pre-dialysis nephrologist as the nephrologist who billed the last inpatient E&M claim within the six months prior to starting dialysis. Because some nephrologists do not manage dialysis and because we were interested in characteristics of nephrologists' primary facilities, we excluded patients who were assigned nephrologists who did not manage dialysis in the same year (i.e., nephrologists who did not bill at least one monthly capitated payment [MCP] dialysis claim) (**eTable 2** for MCP codes). Pre-dialysis nephrologists were not necessarily included during the entirety of the study. If a patient started dialysis during a year when the patient's pre-dialysis nephrologist did not manage any dialysis, that patient was excluded from the study.

A total of 9,140 nephrologists were identified as the pre-dialysis nephrologist in our sample. Approximately 76% of patients had an outpatient pre-dialysis nephrologist and 24% had an inpatient pre-dialysis nephrologist. Patients starting dialysis with an assigned nephrologist who did not manage dialysis comprised only 1.3% of starts.

We identified nephrologists' primary facility as the facility where the nephrologist billed the plurality of dialysis MCP claims in the previous calendar year. Thus, nephrologists could have a different primary facility in each year of our study period. On average, the primary facility constituted 62% of nephrologists' claims. There were 5,161 primary facilities out of the 6,441 total facilities in our sample.

For each pre-dialysis nephrologist, we identified a "regional network." We defined this network as the set of all possible facilities where patients of the nephrologist could choose to start dialysis. We refreshed regional networks for every year that the nephrologist was eligible for inclusion in our study. We first created a preliminary list of facilities that contained every facility where patients of the nephrologist in the current or previous calendar year started dialysis. We added the nephrologist's primary facility to this list. Because our intent was to use these regional networks to create plausible market areas and because this list of facilities does not necessarily include neighboring facilities, we deemed this list of facilities incomplete. Thus, we included all dialysis facilities located in the same zip code as any of the facilities in the preliminary list of facilities. This finalized list of facilities constituted the nephrologist's regional network for that calendar year. **eTable 4** shows characteristics of these networks.

We used this regional network in three ways. First, we used the network to determine whether a facility was close to or far from the patient. We did so because a facility's proximity to the patient is dependent on regional characteristics (e.g., whether the patient lives in a rural area). We show the number of nearby and 4-/5-star facilities available to patients within their regional network in **eTable 5**. In general, most patients had at least two nearby facilities and four high quality facilities in their regional

© 2025 Lin E et al. *JAMA Health Forum*.

network; these numbers did not differ substantially by race/ethnicity. Second, we used the network to estimate each facility's market share because patients have a higher probability of starting dialysis at larger facilities. If nephrologists' primary facilities are also the largest facilities in the regional network, then patients will disproportionately start dialysis at primary facilities simply because these facilities will have more available dialysis spots. Third, we used the network to estimate the Herfindahl-Hirschman Index (HHI) of the market area, so we could control for market consolidation in the regional network. We estimated the HHI for each network-year using the following equation:

$$HHI_{r,y} = \sum_{c} \left(\frac{n_{c,r,y-1}}{N_{r,y-1}} * 100 \right)^2$$

where *r* is the regional network,

y is a specific year,

c is a specific dialysis chain in *r*,

$n_{c,r,y-1}$ is the number of patient months of dialysis in regional network *r*, at chain *c*, in year *y-1*

$N_{r,y-1}$ is the total number of patient months of dialysis in regional network *r* in year *y-1*

and the sum is over all chains *c*.

© 2025 Lin E et al. *JAMA Health Forum*.

eTable 1: Evaluation and Management Codes

HCPCS / CPT	HCPCS/CPT Description
99201	OFFICE/OUTPATIENT VISIT, NEW
99202	OFFICE O/P NEW SF 15-29 MIN
99203	OFFICE O/P NEW LOW 30-44 MIN
99204	OFFICE O/P NEW MOD 45-59 MIN
99205	OFFICE O/P NEW HI 60-74 MIN
99211	OFF/OP EST MAY X REQ PHY/QHP
99212	OFFICE O/P EST SF 10-19 MIN
99213	OFFICE O/P EST LOW 20-29 MIN
99214	OFFICE O/P EST MOD 30-39 MIN
99215	OFFICE O/P EST HI 40-54 MIN
99217	OBSERVATION CARE DISCHARGE
99218	INITIAL OBSERVATION CARE
99219	INITIAL OBSERVATION CARE
99220	INITIAL OBSERVATION CARE
99221	INITIAL HOSPITAL CARE
99222	INITIAL HOSPITAL CARE
99223	INITIAL HOSPITAL CARE
99224	SUBSEQUENT OBSERVATION CARE
99224	UNKNOWN PROCEDURE
99225	SUBSEQUENT OBSERVATION CARE
99225	UNKNOWN PROCEDURE
99226	SUBSEQUENT OBSERVATION CARE
99226	UNKNOWN PROCEDURE
99227	UNKNOWN PROCEDURE
99228	UNKNOWN PROCEDURE
99229	UNKNOWN PROCEDURE
99230	UNKNOWN PROCEDURE
99231	SUBSEQUENT HOSPITAL CARE
99232	SUBSEQUENT HOSPITAL CARE
99233	SUBSEQUENT HOSPITAL CARE
99234	OBSERV/HOSP SAME DATE
99235	OBSERV/HOSP SAME DATE
99236	OBSERV/HOSP SAME DATE
99237	UNKNOWN PROCEDURE
99238	HOSPITAL DISCHARGE DAY
99239	HOSPITAL DISCHARGE DAY
99251	INPATIENT CONSULTATION
99252	INPATIENT CONSULTATION
99253	INPATIENT CONSULTATION
99254	INPATIENT CONSULTATION
99255	INPATIENT CONSULTATION
99281	EMERGENCY DEPT VISIT
99282	EMERGENCY DEPT VISIT
99283	EMERGENCY DEPT VISIT
99284	EMERGENCY DEPT VISIT
99285	EMERGENCY DEPT VISIT
99286	UNKNOWN PROCEDURE
99287	UNKNOWN PROCEDURE
99288	DIRECT ADVANCED LIFE SUPPORT
99291	CRITICAL CARE, FIRST HOUR
99292	CRITICAL CARE, ADDL 30 MIN
99354	PROLNG SVC O/P 1ST HOUR
99355	PROLNG SVC O/P EA ADDL 30
99490	CHRNC CARE MGMT STAFF 1ST 20
99490	UNKNOWN PROCEDURE
99495	TRANS CARE MGMT 14 DAY DISCH
99495	UNKNOWN PROCEDURE
99498	ADVNCD CARE PLAN ADDL 30 MIN
99498	UNKNOWN PROCEDURE
G0402	INIT PREV PE LTD DUR 1ST 12 MOS MCR
G0402	UNKNOWN PROCEDURE
G0438	ANNUAL WELLNES VST; PERSNL PPS INIT
G0438	UNKNOWN PROCEDURE
G0439	ANNUAL WELLNESS VST; PPS SUBSQT VST
G0439	UNKNOWN PROCEDURE

© 2025 Lin E et al. *JAMA Health Forum*.

eTable 2: Monthly Capitated Payment Dialysis Codes

HCPCS / CPT	HCPCS/CPT Description
90951	In-center dialysis, < 2 yrs old, 4+ visits/month
90952	In-center dialysis, < 2 yrs old, 2-3 visits/month
90953	In-center dialysis, < 2 yrs old, 1 visits/month
90954	In-center dialysis, 2-11 yrs old, 4+ visits/month
90955	In-center dialysis, 2-11 yrs old, 2-3 visits/month
90956	In-center dialysis, 2-11 yrs old, 1 visits/month
90957	In-center dialysis, 12-19 yrs old, 4+ visits/month
90958	In-center dialysis, 12-19 yrs old, 2-3 visits/month
90959	In-center dialysis, 12-19 yrs old, 1 visits/month
90960	In-center dialysis, 20+ yrs old, 4+ visits/month
90961	In-center dialysis, 20+ yrs old, 2-3 visits/month
90962	In-center dialysis, 20+ yrs old, 1 visits/month
90963	Home dialysis, < 2 yrs old, monthly visit
90964	Home dialysis, 2-11 yrs old, monthly visit
90965	Home dialysis, 12-19 yrs old, monthly visit
90966	Home dialysis, 20+ yrs old, monthly visit
90967	Home dialysis, < 2 yrs old, partial month
90968	Home dialysis, 2-11 yrs old, partial month
90969	Home dialysis, 12-19 yrs old, partial month
90970	Home dialysis, 20+ yrs old, partial month

Facility Distance to the Patient

Facility distance to the patient should be estimated in relative terms based on the geographic density of the patient's neighborhood. Patients living in rural neighborhoods likely think of geographic proximity on different terms from patients living in urban neighborhoods. Thus, we classified facilities as close to or far from a patient relative to the surrounding facilities in the patient's neighborhood. Below, we describe the methodology for determining whether a facility was "close" to the patient.

As discussed above, we paired each patient with a pre-dialysis nephrologist. For each patient-nephrologist pair, we calculated the distances between the patient's residence and all facilities in the nephrologist's regional network. We had access to facility street addresses but only patient residence zip codes. Thus, we estimated the geodesic distance between the population weighted centroid of the patient's residential zip code to the geocoded longitude and latitude of each facility's street address. We geocoded street addresses using Google Maps' API (Application Programming Interface).[10]

We classified a facility as "close to the patient" if it was in the bottom quartile of distance from the patient's residence among all facilities in the network. When ties in distances resulted in more than 25% of facilities classified as "close," we reclassified these edge cases as distant facilities. In other words, a patient-regional network pair could not classify more than 25% of facilities in a network as "close." In rare circumstances, excluding ties resulted in zero close facilities for that network. For those networks, we considered all facilities as close. From this calculation, we determined whether the patient's starting facility and whether the managing nephrologist's primary facility was close to the patient.

© 2025 Lin E et al. *JAMA Health Forum*.

We further summarized facility distances in the regional network by calculating the bottom 20th, 25th, and 30th percentile distances between patient and facility, when stratifying patients by whether they lived in a rural, micropolitan, or metropolitan zip code (**eTable 6**).

Because this definition of "close" is arbitrary, we conducted a large number of sensitivity analyses to ensure our results were robust to other definitions of close (**eTables 15-16**). We articulate those sensitivity analyses below.

Adjusting for Market Share when Examining Outcomes of the Starting Dialysis Facility

We assessed three outcomes on characteristics of the starting facility: (1) whether the patient's starting facility was the nephrologist's own primary facility, (2) whether the starting facility was high quality (i.e., had a four- or five-star rating), and (3) whether the starting facility was close to the patient.

For these outcomes, we adjusted for regional market share as a potential confounder because primary facilities are likely larger and more likely to be patients' starting facilities even if dialysis starts are completely random. To estimate the regional market share of a facility, we took the total number of dialysis patient-months in the previous year provided by the facility of interest and divided by the total number of dialysis patient-months provided by all facilities with the regional network. We used the previous year's market share to avoid simultaneity bias from dialysis starts impacting market share.

Outcome 1 (primary facility starts) adjusted for the regional market share of the primary facility. Outcomes 2 (four- or five-star facility starts) and 3 (close facility starts) incorporated the total market share of *all four- or five-star facilities* and *all close facilities* in the regional network, respectively.

Adjusting for the Covid-19 Pandemic

We obtained estimates of the severity of Covid-19 at a county-week level from the Centers for Disease Control.[11] We adjusted for new Covid cases and new Covid deaths for patients who initiated dialysis after the pandemic. Because these data were raw counts and did not adjust for the county's population, we divided these counts by county-year population estimates from the American Community Survey.

Because hospitalization and mortality rates were likely influenced by the pandemic, for patients who started dialysis prior to the pandemic but whose first year of dialysis overlapped with the pandemic, we also adjusted for the total number of weeks in the first year of dialysis that coincided with the pandemic. We did not account for "future" regional Covid severity to avoid simultaneity bias.

We conducted sensitivity analyses where we only examined patients who started dialysis prior to the pandemic and did not experience the pandemic during the first year of dialysis (i.e., patients who started dialysis 2015-2018).

Missing Data

We had the following sources of missing data: missing primary facilities for the pre-dialysis nephrologist, missing zip codes (either missing patient residences or missing facility locations), and missing 5-star DFC ratings. In all cases, we opted to exclude patients with missing data rather than conduct an imputation for the following reasons.

The pre-dialysis nephrologist's primary facility was missing when the nephrologist did not manage Medicare patients on dialysis or (probably more common) when the nephrologist only took care

© 2025 Lin E et al. *JAMA Health Forum*.

of patients pre-dialysis. Because these nephrologists were not representative of the entire population, and because it would be inappropriate to impute a primary facility when the nephrologist does not manage dialysis, we did not attempt to impute the nephrologist's primary facility.

Additionally, the important outcomes and exposures for this analysis are contingent on where the patient and dialysis facilities are located. We chose to exclude these patients rather than conduct an imputation because for these patients, the outcome, exposure, and key covariates were all necessarily missing.

Finally, if a facility had a missing 5-star rating, that necessarily implies that the facility did not have an assigned 5-star rating. That is, this measure of facility quality was not transparent to the patient or the nephrologist at the point of decision. Because a key aspect of our analysis was examining decision-making when this information is available to the decision-makers, we did not include them in the analysis.

Sensitivity Analyses

For analyses on starts at nephrologists' primary facilities (**Table 2**), starts at high-quality facilities, and starts at close facilities, we conducted the following sensitivity analyses (**eTables 11-12**):

1) Varied the assignment criteria for the pre-dialysis nephrologist
    a. Restricted to patients with an outpatient nephrologist
    b. Restricted to patients with an inpatient pre-dialysis nephrologist
    c. Reclassified the pre-dialysis nephrologist as the nephrologist billing the last E&M code (outpatient or inpatient)
2) Varied the level of geographic fixed effects
    a. Used hospital referral region (HRR) fixed effects instead of Hospital Service Area (HSA) fixed effects
    b. Divided each HSA into rural, micropolitan, and metropolitan regions using county designations, then used HSA-rural/urbanicity fixed effects
3) Restricted to specific subgroups of interest
    a. Those starting dialysis prior to the Covid-19 pandemic (2015-2018)
    b. Only patients living in metropolitan areas (we could not perform subgroup analyses for patients living in micropolitan or rural areas owing to small sample size)
    c. Only patients starting in-center hemodialysis (we could not perform subgroup analyses for patients starting home dialysis owing to small sample size)
    d. Only patients with 2+ close facilities in regional network
    e. Only patients with 2+ close facilities (with at least 1 of these high quality) in regional network
4) Varied market share controls
    a. Omitted market share adjustments
    b. Subtracted market share from the outcome and omitted market share from the right-hand side

© 2025 Lin E et al. *JAMA Health Forum*.

For analyses on mortality and hospitalization rates, we conducted sensitivity analyses 1-3 and incorporated the following additional analyses (**eTables 13-14**):

5) Varied assumptions on how rates were estimated
    a. Omitted the first 90 days of dialysis when estimating mortality and hospitalization rates (in this analysis, we required that patients receive dialysis for 120 days or more)
    b. Required that patients have at least 30 days of dialysis to contribute to mortality and hospitalization outcomes
    c. Required that patients have at least 90 days of dialysis to contribute to mortality and hospitalization outcomes
    d. Instead of using Poisson regression, we used a weighted linear regression, where the outcome was the number of events divided by the number of days of follow-up, and observations were weighted by the follow-up.

We further tested the sensitivity of our analyses using different methods for measuring the distance between patients and dialysis facilities and using different cutoffs for determining whether a facility was "close" to a patient (**eTables 15-16**). Note, the base case measured the geodesic distance between the population weighted centroid of the patient's residential zip code and the geocoded street address of the dialysis facility. Close facilities were defined as facilities in the bottom 25th percentile of distance. We thus conducted the following sensitivity analyses:

6) Varied definitions of how we determined if a facility was "close" to the patient:
    a. Used the same method to measure distance, but used a 20% cutoff
    b. Used the same method to measure distance, but used a 30% cutoff
    c. Used the same method to measure distance, but determined the 25th percentile distance for all rural, micropolitan, and metropolitan regions. If a facility was within this distance, it was considered "close." (See eTable 6 for kilometer cutoffs).
    d. Distance was measured as the geodesic distance between the geographic centroid of the patient's residential zip code and the facility street address. Used a 25% cutoff.
    e. Distance was measured as the geodesic distance between the population weighted centroid of the patient's residential zip code and the facility zip code. Used a 25% cutoff.
    f. Distance was measured as the geodesic distance between the geographic centroid of the patient's residential zip code and the facility zip code. Used a 25% cutoff.

For analyses on disparities between Black and White patients (**Table 3**), we conducted sensitivity analyses 1-3 and incorporated the following additional analyses (**eTable 17**):

7) Adjusted for the market share of four- and five-star facilities in the regional network (when examining the probability of being referred to a four- or five-star facility)

© 2025 Lin E et al. *JAMA Health Forum*.

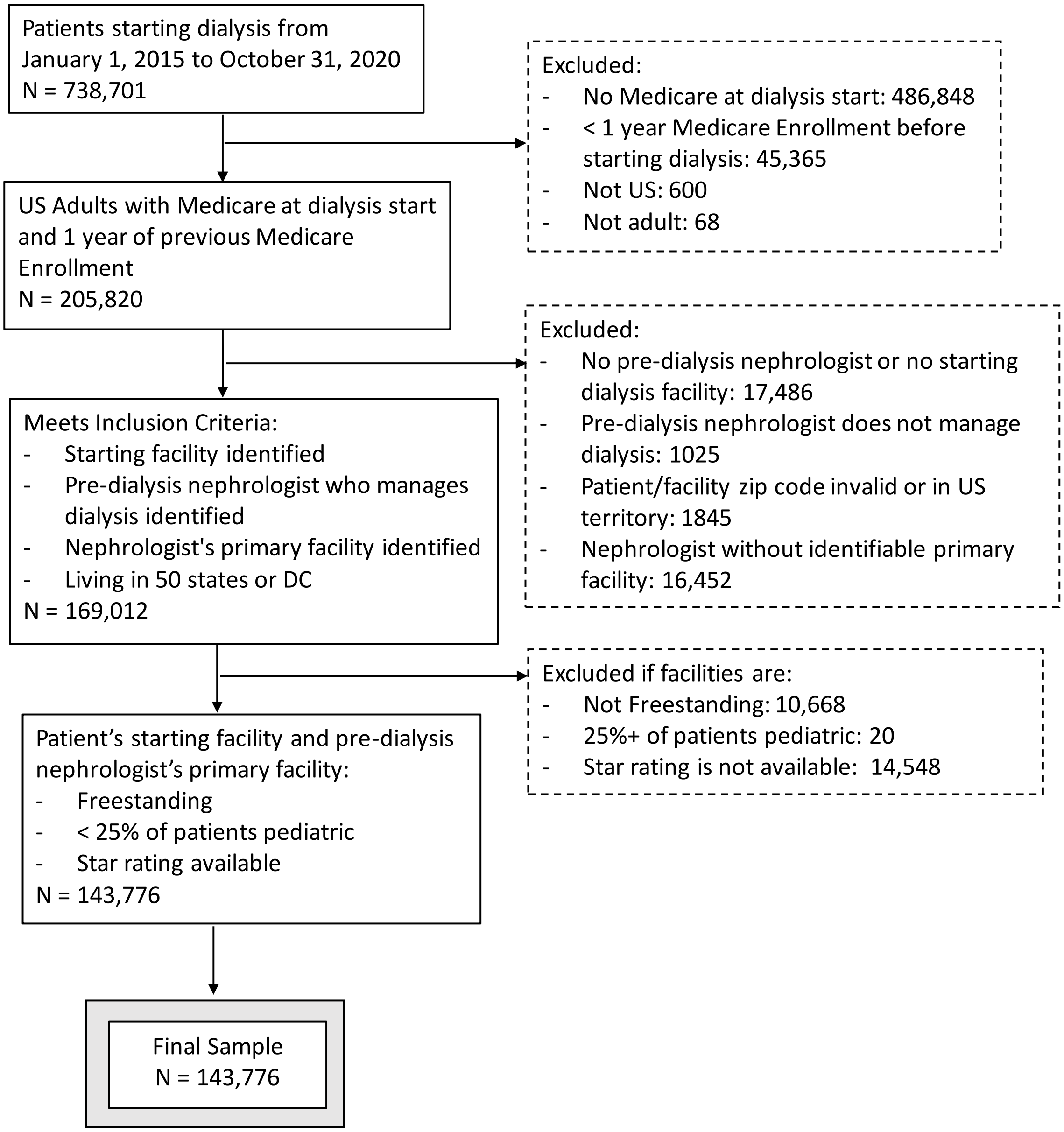

eFigure: CONSORT Diagram

© 2025 Lin E et al. *JAMA Health Forum*.

eTable 3: Mean Characteristics of Pediatric versus Non-Pediatric Facilities

Characteristic	Facility with < 25% Pediatric Patients	Facility with 25%+ Pediatric Patients
# Patients	107	26
# Patient-Months	944	221
% Patients Pediatric	1.1%	81.2%
% Patient-Months Pediatric	1.2%	81.3%
Patient : Staff Ratio	6.1	2.4
% For-Profit, Chain	82.1%	9.5%
% For-Profit, Independent	7.0%	4.2%
% Non-Profit	8.9%	48.2%
% Unknown Profit Status	1.9%	37.8%

Source: Authors' analysis of United States Renal Data System data

© 2025 Lin E et al. *JAMA Health Forum*.

eTable 4: Mean Characteristics of Regional Networks

Characteristic	Rural	Metropolitan
# Zip Codes	6	6
# Facilities	8	9
Maximum Distance Between Any Patient Living in the Regional Network to Any Facility in the Regional Network (Kilometers)	82.2	37.9

Source: Authors' analysis of United States Renal Data System data

© 2025 Lin E et al. *JAMA Health Forum*.

eTable 5: Number of Close and 4- or 5-Star Facilities within Patients' Regional Networks

Race/Ethnicity	# Close Facilities in Region				# 4- and 5-Star Facilities in Region			
	Mean	Median	25th Percentile	75th Percentile	Mean	Median	25th Percentile	75th Percentile
**All Patients**	**2.6**	**2**	**1**	**3**	**5.2**	**4**	**2**	**7**
Non-Hispanic, White	2.5	2	1	3	5.1	4	2	7
Hispanic	2.3	2	1	3	5	4	2	7
Black	3	2	1	4	5.5	5	2	8
Asian	2.3	1	2	3	5.5	5	2	8
Other	2.2	2	1	3	5.4	5	3	7

Source: Authors' analysis of United States Renal Data System data

© 2025 Lin E et al. *JAMA Health Forum.*

eTable 6: The 20th, 25th, and 30th Percentile Distances (in kilometers) between Patients and Dialysis Facilities in their Regional Network, Stratified by Population Density

Density of Zip Code	20th Percentile	25th Percentile	30th Percentile
Metropolitan	7	8	10
Micropolitan	25	32	38
Rural	39	45	51

Source: Authors' analysis of United States Renal Data System data

© 2025 Lin E et al. *JAMA Health Forum*.

eTable 7: Patient Comorbid Conditions, Stratified by Their Pre-Dialysis Nephrologists' Primary Facilities

Characteristic	Referring Nephrologists' Primary Facility											
	Close		Far		p value	Low Quality (1-2 Stars)		Average Quality (3 Stars)		High Quality (4-5 Stars)		p value [a]
	n = 44,978		n = 98,798			n = 22,539		n = 57,051		n = 64,186		
	N / Mean	% / (SD)	N / Mean	% / (SD)		N / Mean	% / (SD)	N / Mean	% / (SD)	N / Mean	% / (SD)	
**Comorbid Conditions**												
Diabetes	32,851	73.0%	72,028	72.9%	0.60	16,426	72.9%	41,623	73.0%	46,830	73.0%	0.97
Congestive Heart Failure	30,575	68.0%	67,264	68.1%	0.69	15,711	69.7%	38,916	68.2%	43,212	67.3%	< 0.001
Hyperlipidemia	32,580	72.4%	71,581	72.5%	0.95	14,287	63.4%	41,329	72.4%	48,545	75.6%	< 0.001
Hypertension	43,117	95.9%	94,849	96.0%	0.21	21,010	93.2%	54,619	95.7%	62,337	97.1%	< 0.001
Ischemic Heart Disease	29,098	64.7%	64,162	64.9%	0.36	15,026	66.7%	37,094	65.0%	41,140	64.1%	< 0.001
Anemia	42,211	93.8%	92,946	94.1%	0.09	20,774	92.2%	53,592	93.9%	60,791	94.7%	< 0.001
Acquired Hypothyroidism	10,390	23.1%	22,795	23.1%	0.91	4,229	18.8%	12,873	22.6%	16,083	25.1%	< 0.001
Alzheimer's Disease	1,077	2.4%	2,492	2.5%	0.15	647	2.9%	1,441	2.5%	1,481	2.3%	< 0.001
Asthma	13,622	30.3%	30,259	30.6%	0.19	6,077	27.0%	17,702	31.0%	20,102	31.3%	< 0.001
Alzheimer's Disease and Related Disorders or Senile Dementia	5,942	13.2%	13,817	14.0%	< 0.001	3,130	13.9%	7,893	13.8%	8,736	13.6%	0.42
Atrial Fibrillation	14,266	31.7%	31,129	31.5%	0.43	7,155	31.7%	18,067	31.7%	20,173	31.4%	0.56
Acute Myocardial Infarction	4,498	10.0%	10,236	10.4%	0.04	2,533	11.2%	5,970	10.5%	6,231	9.7%	< 0.001
Female Breast Cancer	1,236	2.7%	2,767	2.8%	0.57	495	2.2%	1,691	3.0%	1,817	2.8%	< 0.001
Cataract	8,383	18.6%	18,642	18.9%	0.30	4,037	17.9%	10,586	18.6%	12,402	19.3%	< 0.001
Chronic Obstructive Pulmonary Disease	15,283	34.0%	33,375	33.8%	0.46	8,117	36.0%	19,750	34.6%	20,791	32.4%	< 0.001
Colorectal Cancer	1,257	2.8%	2,794	2.8%	0.72	590	2.6%	1,644	2.9%	1,817	2.8%	0.12
Depression	13,932	31.0%	30,956	31.3%	0.18	7,100	31.5%	17,641	30.9%	20,147	31.4%	0.13
Endometrial Cancer	364	0.8%	747	0.8%	0.29	164	0.7%	481	0.8%	466	0.7%	0.05
Glaucoma	5,252	11.7%	11,433	11.6%	0.57	2,515	11.2%	6,722	11.8%	7,448	11.6%	0.05
Hip/Pelvic Fracture	547	1.2%	1,191	1.2%	0.86	278	1.2%	694	1.2%	766	1.2%	0.87
Lung Cancer	605	1.3%	1,295	1.3%	0.60	276	1.2%	767	1.3%	857	1.3%	0.38
Osteoporosis	3,306	7.4%	7,059	7.1%	0.16	1,663	7.4%	4,059	7.1%	4,643	7.2%	0.41
Prostate Cancer	2,513	5.6%	5,738	5.8%	0.10	1,256	5.6%	3,250	5.7%	3,745	5.8%	0.30

© 2025 Lin E et al. *JAMA Health Forum.*

eTable 7: Patient Comorbid Conditions, Stratified by Their Pre-Dialysis Nephrologists' Primary Facilities, Continued

Characteristic	Referring Nephrologists' Primary Facility												
	Close		Far		p value	Low Quality (1-2 Stars)		Average Quality (3 Stars)		High Quality (4-5 Stars)		p value [a]
	n = 44,978		n = 98,798			n = 22,539		n = 57,051		n = 64,186		
	N / Mean	% / (SD)	N / Mean	% / (SD)		N / Mean	% / (SD)	N / Mean	% / (SD)	N / Mean	% / (SD)	
**Comorbid Conditions (continued)**												
Rheumatoid Arthritis/Osteoarthritis	14,843	33.0%	32,676	33.1%	0.79	7,066	31.4%	19,009	33.3%	21,444	33.4%	< 0.001
Stroke/Transient Ischemic Attack	5,279	11.7%	12,136	12.3%	< 0.01	2,866	12.7%	7,094	12.4%	7,455	11.6%	< 0.001
Benign Prostatic Hypertrophy	9,311	20.7%	20,297	20.5%	0.49	4,453	19.8%	11,533	20.2%	13,622	21.2%	< 0.001
**Season of Year**												
Winter	11,543	25.7%	25,036	25.3%	0.04	5,716	25.4%	14,632	25.6%	16,231	25.3%	0.12
Spring	12,238	27.2%	26,447	26.8%		6,081	27.0%	15,292	26.8%	17,312	27.0%	
Summer	11,067	24.6%	24,904	25.2%		5,527	24.5%	14,207	24.9%	16,237	25.3%	
Fall	10,130	22.5%	22,411	22.7%		5,215	23.1%	12,920	22.6%	14,406	22.4%	

Abbreviations: SD = standard deviation;
Notes: For continuous variables, the standard deviation is shown in parentheses.
[a] p-values computed using the Pearson's chi-square test for categorical variables and the Kruskal-Wallis test for continuous variables.
Source: Authors' analysis of United States Renal Data System data

© 2025 Lin E et al. *JAMA Health Forum*.

eTable 8: Unadjusted Probabilities of Starting at the Nephrologist's Primary Facility and of Starting at a 4- or 5-Star Facility

Nephrologist's Primary Facility is:	Outcome: Starting at Primary Facility				Outcome: Starting at 4- or 5-Star Facility			
	% Starting at Primary Facility	% Market Share of Primary Facility	% Excess over Market Share		% Starting at 4- or 5-Star Facility	% Market Share of 4- or 5-Star Facilities	% Excess over Market Share	
			%	95% CI			%	95% CI
4- or 5-Star and Close to Patient	65.2%	26.5%	38.8%	38.2%, 39.3%	84.4%	66.7%	17.7%	17.3%, 18.1%
1- or 2-Star and Close to Patient	63.8%	24.6%	39.1%	38.2%, 40.0%	7.3%	18.9%	-11.7%	-12.2%, -11.1%
4- or 5-Star and Far from Patient	20.5%	18.4%	2.0%	1.7%, 2.3%	66.5%	63.3%	3.3%	2.9%, 3.6%
1- or 2-Star and Far from Patient	24.0%	18.4%	5.5%	5.0%, 6.1%	18.0%	20.0%	-2.0%	-2.5%, -1.6%

Notes: These data correspond to Figure 1a-1b.
Source: Authors' analysis of United States Renal Data System data

© 2025 Lin E et al. *JAMA Health Forum*.

eTable 9: Unadjusted Person-Year Rates of Mortality and Hospitalization, Stratified by the Quality of the Nephrologist's Primary Facility and the Starting Facility

Nephrologist's Primary Facility is:	Mortality Rate (per 100 patient-years)		Hospitalization Rate (per 100 patient-years)	
	Rate	95% CI	Rate	95% CI
4- or 5-Star	29.9	29.4, 30.4	173	172, 175
1- or 2 Star	33.1	32.3, 34.1	209	205, 212
**Starting Facility is:**				
4- or 5-Star	29.1	28.6, 29.5	170	168, 171
1- or 2 Star	36.3	35.5, 37.2	217	214, 220

Notes: These data correspond to Figure 1c-1d.
Source: Authors' analysis of United States Renal Data System data

© 2025 Lin E et al. *JAMA Health Forum*.

eTable 10: Unadjusted Probability of Having a Nephrologist with a High-Quality Primary Facility and of Starting at a High-Quality Facility, Stratified by Race

Patinet's Race/Ethnicity	Nephrologist's Primary Facility is 4- or 5- Stars		Starting Facility is 4- or 5-Stars	
	%	95% CI	%	95% CI
White, Non-Hispanic	45.2%	45.0%, 45.5%	46.2%	45.9%, 46.5%
Hispanic	50.5%	49.8%, 51.3%	47.8%	47.0%, 48.5%
Black	37.3%	36.8%, 37.8%	36.0%	35.5%, 36.5%
Asian	55.6%	54.4%, 56.8%	53.2%	52.1%, 54.4%
Other	57.2%	55.5%, 58.9%	60.5%	58.8%, 62.1%

Notes: These data correspond to Figure 2.
Source: Authors' analysis of United States Renal Data System data

© 2025 Lin E et al. *JAMA Health Forum*.

eTable 11: Sensitivity Analysis of Starting Facility Outcomes, Comparing Nephrologists' Primary Facilities that are Nearby to Primary Facilities that are Far from Patient

Analysis	Change in Starting Facility Outcomes					
	Starting at Primary Facility		Starting at Close Facility		Starting at 4- or 5-Star Facility	
	P.P. Change	95% CI	P.P. Change	95% CI	P.P. Change	95% CI
**Base Case**	**33.9%**	**33.0%, 34.9%**	**20.2%**	**19.2%, 21.2%**	**-0.3%**	**-0.9%, 0.2%**
1a: Only patients with outpatient referring nephrologist	34.5%	33.5%, 35.4%	22.5%	21.4%, 23.5%	-0.3%	-1.0%, 0.3%
1b: Only patients with inpatient referring nephrologist	20.4%	19.5%, 21.3%	16.3%	15.2%, 17.3%	-0.5%	-1.2%, 0.1%
1c: Referring nephrologist classified as the nephrologist billing the last E/M	27.0%	26.2%, 27.8%	18.5%	17.6%, 19.5%	-0.4%	-1.0%, 0.2%
2a: Using HRR level fixed effects	35.8%	34.4%, 37.3%	19.7%	18.3%, 21.1%	-0.7%	-1.4%, -0.1%
2b: Using HSA-urbanicity fixed effects, where HSAs are subdivided into rural, micropolitan, and metropolitan regions	33.9%	33.0%, 34.8%	20.3%	19.3%, 21.3%	-0.3%	-0.8%, 0.3%
3a: Pre-Covid (Patients starting 2015-2018)	34.1%	33.0%, 35.2%	20.3%	19.3%, 21.4%	-0.6%	-1.3%, 0.0%
3b: Only patients in metropolitan areas	32.0%	31.0%, 33.0%	21.9%	20.8%, 23.0%	0.1%	-0.5%, 0.8%
3c: Only patients starting in-center hemodialysis	35.5%	34.4%, 36.5%	19.4%	18.3%, 20.4%	-0.3%	-0.9%, 0.3%
3d: Only patients with 2+ close facilities in region	32.5%	31.4%, 33.6%	17.1%	16.0%, 18.2%	-0.5%	-1.2%, 0.2%
3e: Only patients with 2+ close facilities (with at least 1 high quality) in region	32.1%	30.9%, 33.3%	15.5%	14.3%, 16.7%	0.4%	-0.6%, 1.4%
4a: Omitting market share	37.1%	36.0%, 38.2%	26.7%	25.7%, 27.8%	-0.6%	-1.2%, 0.0%
4b: Subtracting market share from the outcome	33.2%	32.3%, 34.1%	18.1%	17.2%, 19.1%	-0.3%	-0.8%, 0.3%

Abbreviations: p.p. = percentage point; E&M = evaluation and management; HRR = hospital referral region; HSA = Hospital Service Area
Source: Authors' analysis of United States Renal Data System data

© 2025 Lin E et al. *JAMA Health Forum.*

eTable 12: Sensitivity Analysis of Starting Facility Outcomes, Comparing Nephrologists' Primary Facilities that are High Quality to Low Quality

Analysis	Change in Starting Facility Outcomes					
	Starting at Primary Facility		Starting at Close Facility		Starting at 4- or 5-Star Facility	
	P.P. Change	95% CI	P.P. Change	95% CI	P.P. Change	95% CI
**Base Case**	**-0.5%**	**-0.8%, -0.1%**	**0.4%**	**0.1%, 0.8%**	**7.4%**	**6.9%, 7.9%**
1a: Only patients with outpatient referring nephrologist	-0.5%	-0.9%, 0.0%	0.5%	0.1%, 0.9%	8.3%	7.8%, 8.9%
1b: Only patients with inpatient referring nephrologist	-0.4%	-0.9%, 0.0%	0.2%	-0.3%, 0.6%	8.0%	7.4%, 8.6%
1c: Referring nephrologist classified as the nephrologist billing the last E/M	-0.3%	-0.7%, 0.1%	0.5%	0.1%, 0.8%	8.1%	7.5%, 8.6%
2a: Using HRR level fixed effects	-0.5%	-1.0%, 0.0%	0.4%	0.1%, 0.8%	8.1%	7.5%, 8.7%
2b: Using HSA-urbanicity fixed effects, where HSAs are subdivided into rural, micropolitan, and metropolitan regions	-0.5%	-0.9%, -0.1%	0.4%	0.0%, 0.7%	7.5%	7.0%, 8.0%
3a: Pre-Covid (Patients starting 2015-2018)	-0.4%	-0.8%, 0.1%	0.4%	0.0%, 0.8%	6.9%	6.4%, 7.5%
3b: Only patients in metropolitan areas	-0.7%	-1.1%, -0.3%	0.5%	0.1%, 0.9%	7.9%	7.4%, 8.5%
3c: Only patients starting in-center hemodialysis	0.0%	-0.4%, 0.3%	0.3%	0.0%, 0.7%	7.0%	6.5%, 7.5%
3d: Only patients with 2+ close facilities in region	-0.4%	-0.8%, 0.0%	0.4%	0.0%, 0.8%	7.0%	6.5%, 7.6%
3e: Only patients with 2+ close facilities (with at least 1 high quality) in region	0.0%	-0.5%, 0.5%	0.4%	-0.2%, 0.9%	7.3%	6.6%, 8.0%
4a: Omitting market share	-0.9%	-1.3%, -0.5%	0.3%	-0.1%, 0.7%	14.1%	13.5%, 14.6%
4b: Subtracting market share from the outcome	-0.4%	-0.7%, 0.0%	0.4%	0.1%, 0.8%	4.8%	4.3%, 5.3%

Notes: Estimates are percentage point changes when the quality of the nephrologist's primary facility increases by 1-star
Abbreviations: p.p. = percentage point; E&M = evaluation and management; HRR = hospital referral region; HSA = Hospital Service Area
Source: Authors' analysis of United States Renal Data System data

© 2025 Lin E et al. *JAMA Health Forum.*

eTable 13: Sensitivity Analysis of Mortality and Hospitalization Outcomes, Comparing Nephrologists' Primary Facilities that are Nearby to Primary Facilities that are Far from Patient

Analysis	Change in Mortality / Hospitalization			
	Mortality (IRR)		Hospitalizations (IRR)	
	P.P. Change	95% CI	P.P. Change	95% CI
**Base Case (IRR)**	**1.044**	**1.019, 1.069**	**0.986**	**0.971, 1.001**
1a: Only patients with outpatient referring nephrologist (IRR)	1.074	1.043, 1.105	1.000	0.982, 1.017
1b: Only patients with inpatient referring nephrologist (IRR)	1.056	1.026, 1.087	0.999	0.980, 1.018
1c: Referring nephrologist classified as the nephrologist billing the last E/M (IRR)	1.035	1.008, 1.063	0.987	0.971, 1.003
2a: Using HRR level fixed effects (IRR)	1.044	1.022, 1.066	0.987	0.972, 1.002
2b: Using HSA-urbanicity fixed effects, where HSAs are subdivided into rural, micropolitan, and metropolitan regions (IRR)	1.045	1.019, 1.071	0.986	0.971, 1.001
3a: Pre-Covid (Patients starting 2015-2018) (IRR)	1.056	1.026, 1.086	0.985	0.968, 1.002
3b: Only patients in metropolitan areas (IRR)	1.053	1.025, 1.082	0.991	0.974, 1.007
3c: Only patients starting in-center hemodialysis (IRR)	1.047	1.021, 1.073	0.986	0.971, 1.002
3d: Only patients with 2+ close facilities in region (IRR)	1.041	1.011, 1.072	0.984	0.966, 1.003
3e: Only patients with 2+ close facilities (with at least 1 high quality) in region (IRR)	1.040	1.001, 1.081	0.972	0.950, 0.995
5a: Omitting first 90 days of dialysis (IRR)	1.048	1.019, 1.077	0.984	0.967, 1.002
5b: Requiring 30+ days of dialysis (IRR)	1.042	1.016, 1.068	0.985	0.970, 1.000
5c: Requiring 90+ days of dialysis (IRR)	1.049	1.021, 1.077	0.985	0.970, 1.001
**Base Case (Rate per 100 Person-Years)**	**1.33**	**0.56, 2.11**	**-2.63**	**-5.39, 0.17**
5d: Weighted linear regression (Rate per 100 Person-Years)	1.27	0.52, 2.03	-2.53	-5.32, 0.25

Notes: Estimates for all analyses are incidence rate ratios (IRRs) obtained from Poisson regression. Analysis 5d shows percentage point changes in the person-year rate of mortality and hospitalization, estimated from a weighted linear regression.
Abbreviations: p.p. = percentage point; E&M = evaluation and management; HRR = hospital referral region; HSA = Hospital Service Area; IRR = incidence rate ratio
Source: Authors' analysis of United States Renal Data System data

© 2025 Lin E et al. *JAMA Health Forum.*

eTable 14: Sensitivity Analysis of Mortality and Hospitalization Outcomes, Comparing Primary Facilities that are High Quality to Low Quality

Analysis	Change in Mortality / Hospitalization			
	Mortality (IRR)		Hospitalizations (IRR)	
	P.P. Change	95% CI	P.P. Change	95% CI
**Base Case (IRR)**	**0.988**	**0.975, 1.002**	**0.979**	**0.972, 0.987**
1a: Only patients with outpatient referring nephrologist (IRR)	0.997	0.982, 1.013	0.982	0.972, 0.991
1b: Only patients with inpatient referring nephrologist (IRR)	0.995	0.979, 1.011	0.982	0.972, 0.992
1c: Referring nephrologist classified as the nephrologist billing the last E/M (IRR)	0.986	0.973, 1.000	0.981	0.973, 0.989
2a: Using HRR level fixed effects (IRR)	0.984	0.971, 0.996	0.974	0.966, 0.981
2b: Using HSA-urbanicity fixed effects, where HSAs are subdivided into rural, micropolitan, and metropolitan regions (IRR)	0.988	0.975, 1.001	0.979	0.971, 0.987
3a: Pre-Covid (Patients starting 2015-2018) (IRR)	0.983	0.968, 0.998	0.981	0.972, 0.990
3b: Only patients in metropolitan areas (IRR)	0.988	0.974, 1.003	0.980	0.971, 0.989
3c: Only patients starting in-center hemodialysis (IRR)	0.989	0.976, 1.003	0.978	0.970, 0.986
3d: Only patients with 2+ close facilities in region (IRR)	0.987	0.971, 1.002	0.981	0.971, 0.990
3e: Only patients with 2+ close facilities (with at least 1 high quality) in region (IRR)	0.991	0.971, 1.012	0.974	0.962, 0.985
5a: Omitting first 90 days of dialysis (IRR)	0.990	0.974, 1.006	0.975	0.966, 0.985
5b: Requiring 30+ days of dialysis (IRR)	0.987	0.974, 1.001	0.979	0.971, 0.987
5c: Requiring 90+ days of dialysis (IRR)	0.990	0.975, 1.006	0.978	0.970, 0.987
**Base Case (Rate per 100 Person-Years)**	**-0.40**	**-0.85, 0.05**	**-4.48**	**-6.12, -2.82**
5d: Weighted linear regression (Rate per 100 Person-Years)	-0.40	-0.81, 0.01	-4.09	-5.60, -2.59

Notes: Estimates are percentage point changes when the quality of the nephrologist's primary facility increases by 1-star. Estimates for all analyses are incidence rate ratios (IRRs) obtained from Poisson regression. Analysis 5d shows percentage point changes in the person-year rate of mortality and hospitalization, estimated from a weighted linear regression.
Abbreviations: p.p. = percentage point; E&M = evaluation and management; HRR = hospital referral region; HSA = Hospital Service Area; IRR = incidence rate ratio
Source: Authors' analysis of United States Renal Data System data

© 2025 Lin E et al. *JAMA Health Forum.*

eTable 15: Sensitivity Analysis of Starting Facility Outcomes, Comparing Primary Facilities that are Close to Facilities that are Far from the Patient, Using Different Definitions of Distance

Analysis	Change in Starting Facility Outcomes					
	Starting at Primary Facility		Starting at Close Facility		Starting at 4- or 5-Star Facility	
	P.P. Change	95% CI	P.P. Change	95% CI	P.P. Change	95% CI
**Base Case: Distance measured using population weighted centroid of patient zip code to facility street address (25% cutoff = close)**	**33.9%**	**33.0%, 34.9%**	**20.2%**	**19.2%, 21.2%**	**-0.3%**	**-0.9%, 0.2%**
6a: Base Case but 20% cutoff = close	36.4%	35.4%, 37.4%	24.5%	23.4%, 25.6%	-0.6%	-1.1%, 0.0%
6b: Base Case but 30% cutoff = close	32.2%	31.3%, 33.1%	20.9%	19.9%, 21.8%	-0.2%	-0.7%, 0.4%
6c: Base Case but close facilities defined as patients traveling < 25th percentile, stratified by rural, micropolitan, and metropolitan regions	34.1%	33.0%, 35.2%	35.9%	34.9%, 36.9%	-0.4%	-1.0%, 0.2%
6d: Distance measured using geographic centroid of patient zip code to facility street address (25% cutoff = close)	33.6%	32.6%, 34.5%	21.1%	20.1%, 22.1%	-0.3%	-0.9%, 0.2%
6e: Distance measured using population weighted centroid of patient zip code to geographic centroid of facility zip code (25% cutoff = close)	37.5%	36.4%, 38.6%	20.7%	19.6%, 21.7%	-0.7%	-1.3%, -0.1%
6f: Distance measured using geographic centroid of patient zip code to graphic centroid of facility zip code (25% cutoff = close)	36.8%	35.7%, 37.9%	20.9%	19.9%, 22.0%	-0.6%	-1.2%, -0.1%

Abbreviations: p.p. = percentage point
Source: Authors' analysis of United States Renal Data System data

© 2025 Lin E et al. *JAMA Health Forum.*

eTable 16: Sensitivity Analysis of Starting Facility Outcomes, Comparing Primary Facilities that are High Quality to Facilities that are Low Quality, Using Different Definitions of Distance

Analysis	Change in Starting Facility Outcomes					
	Starting at Primary Facility		Starting at Close Facility		Starting at 4- or 5-Star Facility	
	P.P. Change	95% CI	P.P. Change	95% CI	P.P. Change	95% CI
**Base Case: Distance measured using population weighted centroid of patient zip code to facility street address (25% cutoff = close)**	**-0.5%**	**-0.8%, -0.1%**	**0.4%**	**0.1%, 0.8%**	**7.4%**	**6.9%, 7.9%**
6a: Base Case but 20% cutoff = close	-0.5%	-0.9%, -0.1%	0.1%	-0.2%, 0.5%	7.4%	6.9%, 7.9%
6b: Base Case but 30% cutoff = close	-0.4%	-0.8%, 0.0%	0.5%	0.2%, 0.8%	7.4%	6.9%, 7.9%
6c: Base Case but close facilities defined as patients traveling < 25th percentile, stratified by rural, micropolitan, and metropolitan regions	-0.4%	-0.7%, 0.0%	0.5%	0.2%, 0.8%	7.4%	6.9%, 7.9%
6d: Distance measured using geographic centroid of patient zip code to facility street address (25% cutoff = close)	-0.5%	-0.9%, -0.1%	0.3%	0.0%, 0.7%	7.4%	6.9%, 7.9%
6e: Distance measured using population weighted centroid of patient zip code to geographic centroid of facility zip code (25% cutoff = close)	-0.5%	-0.9%, -0.2%	0.4%	0.1%, 0.7%	7.4%	6.9%, 7.9%
6f: Distance measured using geographic centroid of patient zip code to graphic centroid of facility zip code (25% cutoff = close)	-0.5%	-0.8%, -0.1%	0.4%	0.1%, 0.7%	7.4%	6.9%, 7.9%

Abbreviations: p.p. = percentage point
Source: Authors' analysis of United States Renal Data System data

© 2025 Lin E et al. *JAMA Health Forum.*

eTable 17: Sensitivity Analysis of Disparities between Black and White Patients

Analysis	Overall		Fixed Effects Model	
	P.P. Change	95% CI	P.P. Change	95% CI
**Likelihood of Having a Nephrologist with a 4- or 5-Star Primary Facility**				
**Base Case**	**-4.6%**	**-6.0%, -3.2%**	**-2.0%**	**-3.0%, -1.0%**
1a: Only patients with outpatient referring nephrologist	-4.8%	-6.3%, -3.3%	-1.9%	-2.9%, -0.9%
1b: Only patients with inpatient referring nephrologist	-4.4%	-6.0%, -2.8%	-1.8%	-3.1%, -0.5%
1c: Referring nephrologist classified as the nephrologist billing the last E/M	-4.9%	-6.4%, -3.5%	-2.1%	-3.2%, -1.1%
2a: Using HRR level fixed effects	-4.6%	-6.4%, -2.8%	-2.7%	-4.1%, -1.4%
2b: Using HSA-urbanicity fixed effects, where HSAs are subdivided into rural, micropolitan, and metropolitan regions	-4.6%	-6.0%, -3.2%	-2.1%	-3.1%, -1.1%
3a: Pre-Covid (Patients starting 2015-2018)	-4.0%	-5.4%, -2.6%	-1.6%	-2.5%, -0.6%
3b: Only patients in metropolitan areas	-4.9%	-6.5%, -3.3%	-2.4%	-3.5%, -1.3%
3c: Only patients starting in-center hemodialysis	-4.6%	-6.0%, -3.1%	-1.9%	-3.0%, -0.9%
3d: Only patients with 2+ close facilities in referral region	-4.3%	-5.8%, -2.7%	-1.7%	-2.8%, -0.6%
3e: Only patients with 2+ close facilities (with at least 1 high quality) in region	-5.2%	-6.9%, -3.5%	-1.9%	-3.2%, -0.5%
4: Including market share	-4.7%	-6.1%, -3.3%	-2.0%	-3.0%, -1.0%
**Likelihood of Being Referred to a 4- or 5-Star Facility**				
**Base Case**	**-5.8%**	**-7.2%, -4.3%**	**-2.8%**	**-3.9%, -1.7%**
1a: Only patients with outpatient referring nephrologist	-5.7%	-7.2%, -4.1%	-2.6%	-3.8%, -1.4%
1b: Only patients with inpatient referring nephrologist	-5.9%	-7.5%, -4.3%	-2.9%	-4.3%, -1.5%
1c: Referring nephrologist classified as the nephrologist billing the last E/M	-5.7%	-7.2%, -4.2%	-2.5%	-3.7%, -1.3%
2a: Using HRR level fixed effects	-5.8%	-7.6%, -3.9%	-4.0%	-5.4%, -2.6%
2b: Using HSA-urbanicity fixed effects, where HSAs are subdivided into rural, micropolitan, and metropolitan regions	-5.8%	-7.2%, -4.3%	-2.8%	-4.0%, -1.7%
3a: Pre-Covid (Patients starting 2015-2018)	-4.9%	-6.3%, -3.4%	-2.3%	-3.5%, -1.2%
3b: Only patients in metropolitan areas	-6.1%	-7.6%, -4.5%	-3.2%	-4.4%, -1.9%
3c: Only patients starting in-center hemodialysis	-6.1%	-7.6%, -4.6%	-3.2%	-4.4%, -2.0%
3d: Only patients with 2+ close facilities in referral region	-5.8%	-7.4%, -4.3%	-2.8%	-4.0%, -1.6%
3e: Only patients with 2+ close facilities (with at least 1 high quality) in region	-6.7%	-8.4%, -5.0%	-2.3%	-3.8%, -0.8%
4: Including market share	-1.6%	-2.5%, -0.8%	-1.6%	-2.5%, -0.7%

Abbreviations: p.p. = percentage point; E/M = evaluation and management; HRR = hospital referral region; HSA = Hospital Service Area
Source: Authors' analysis of United States Renal Data System data

© 2025 Lin E et al. *JAMA Health Forum.*

eReferences

1. United States Renal Data System. *2020 USRDS Annual Data Report: Epidemiology of Kidney Disease in the United States*. Bethesda, MD: National Institutes of Health, National Institute of Diabetes and Digestive and Kidney Diseases; 2020. Accessed February 3, 2021. https://adr.usrds.org/2020

2. Centers for Medicare and Medicaid Services (CMS), HHS. End Stage Renal Disease Medical Evidence Report: Medicare Entitlement and/or Patient Registration. October 2018. Accessed December 7, 2020. https://www.cms.gov/Medicare/CMS-Forms/CMS-Forms/Downloads/CMS2728.pdf

3. Centers for Medicare and Medicaid Services. End Stage Renal Disease Medical Information System: ESRD Facility Survey (Dialysis Units Only). July 2020. Accessed December 7, 2020. https://www.cms.gov/Medicare/CMS-Forms/CMS-Forms/Downloads/CMS2744A.pdf

4. Centers for Medicare & Medicaid Services. Dialysis Facility Compare datasets. July 22, 2020. Accessed October 9, 2020. https://data.medicare.gov/data/dialysis-facility-compare

5. Centers for Medicare & Medicaid Services. National Plan & Provider Enumeration System. 2021. Accessed September 15, 2021. https://nppes.cms.hhs.gov/#/

6. The United States Census Bureau. The American Community Survey. 2012. https://www.census.gov/programs-surveys/acs/

7. Health Resources and Services Administration, US Department of Health and Human Services. Area Health Resources Files. July 31, 2022. Accessed December 1, 2023. https://data.hrsa.gov/topics/health-workforce/ahrf

8. Dartmouth Medical School Center for the Evaluative Clinical Sciences. Appendix on the Geography of Health Care in the United States. 1996. Accessed September 25, 2022. https://data.dartmouthatlas.org/downloads/methods/geogappdx.pdf

9. Centers for Medicare & Medicaid Services. Chronic Conditions Data Warehouse: Condition Categories. February 2020. Accessed October 9, 2020. https://www2.ccwdata.org/web/guest/condition-categories

10. Google. Google Maps Platform. (n.d.). Online. (n.d.). Accessed December 1, 2024. https://developers.google.com/maps

11. Centers for Disease Control and Prevention. Weekly United States COVID-19 Cases and Deaths by County - ARCHIVED. Published online January 13, 2025. Accessed December 1, 2025. https://data.cdc.gov/dataset/Weekly-United-States-COVID-19-Cases-and-Deaths-by-/yviw-z6j5/about_data

© 2025 Lin E et al. *JAMA Health Forum*.
