# Supplementary material for: Care Continuity, Nephrologists’ Dialysis Facility Preferences, and Outcomes
Source: JAMA Health Forum. 2025 Apr 11;6(4):e250423. doi: 10.1001/jamahealthforum.2025.0423 (PMC11992609; doi:10.1001/jamahealthforum.2025.0423)
Supplement: Supplement 2. — Data Sharing Statement [file jamahealthforum-e250423-s002.pdf]

Data Sharing Statement

Lin. Care Continuity, Nephrologists' Dialysis Facility Preferences, and Outcomes. *JAMA Health Forum*. Published April 11, 2025. doi:10.1001/jamahealthforum.2025.0423

Data
Data available: No

Additional Information
Explanation for why data not available: The data are freely available to those who sign a data use agreement with the United States Renal Data System. We cannot share the data ourselves because of our data use agreement.
